# Nuclear actin assembly is an integral part of decidualization in human endometrial stromal cells

**DOI:** 10.1038/s42003-024-06492-z

**Published:** 2024-07-11

**Authors:** Isao Tamura, Kei Miyamoto, Chiharu Hatanaka, Amon Shiroshita, Taishi Fujimura, Yuichiro Shirafuta, Yumiko Mihara, Ryo Maekawa, Toshiaki Taketani, Shun Sato, Kazuya Matsumoto, Hiroshi Tamura, Norihiro Sugino

**Affiliations:** 1 Department of Obstetrics and Gynecology, Yamaguchi University Graduate School of Medicine, Minamikogushi 1-1-1, Ube, 755-8505 Japan; 2 Laboratory of Molecular Developmental Biology, Faculty of Biology-Oriented Science and Technology, Kindai University, Wakayama, 649-6493 Japan; 3 Present Address: Laboratory of Animal Reproductive Physiology, Faculty of Agriculture, Kyushu University, Fukuoka, 819-0395 Japan

**Keywords:** Molecular biology, Cell biology

## Abstract

Decidualization of the human endometrium is critical for establishing pregnancy and is entailed by differentiation of endometrial stromal cells (ESCs) into decidual cells. During decidualization, the actin cytoskeleton is dynamically reorganized for the ESCs’ morphological and functional changes. Although actin dynamically alters its polymerized state upon external stimuli not only in the cytoplasm, but also in the nucleus, nuclear actin dynamics during decidualization have not been elucidated. Here, we show that nuclear actin was specifically assembled during decidualization of human ESCs. This decidualization-specific formation of nuclear actin filaments was disassembled following the withdrawal of the decidualization stimulus, suggesting its reversible process. Mechanistically, RNA-seq analyses revealed that the forced disassembly of nuclear actin resulted in the suppression of decidualization, accompanied with the abnormal upregulation of cell proliferation genes, leading to incomplete cell cycle arrest. CCAAT/enhancer-binding protein beta (C/EBPβ), an important regulator for decidualization, was responsible for downregulation of the nuclear actin exporter, thus accelerating nuclear actin accumulation and its assembly for decidualization. Taken together, we demonstrate that decidualization-specific nuclear actin assembly induces cell cycle arrest for establishing the decidualized state of ESCs. We propose that not only the cytoplasmic actin, but also nuclear actin dynamics profoundly affect decidualization process in humans for ensuring pregnancy.

## Abstract (web summary)

Nuclear actin assembly occurs during decidualization of human endometrial stromal cells. This contributes to cell differentiation by inducing cell cycle arrest for establishing the decidualized state of endometrial stromal cells.

## Introduction

Human endometrial stromal cells (hESCs) undergo cyclic changes during the menstrual cycle in response to changing levels of steroid hormones. Especially, hESCs morphologically and functionally change their cellular states for preparing pregnancy, referred to as decidualization. Decidualization is characterized by the process in which hESCs differentiate into decidual cells by the action of progesterone, and is essential for implantation and maintenance of pregnancy^1–3^. The spatiotemporal regulation of decidualization is important for the successful establishment of pregnancy because the decidua is thought to play a key role in regulating trophoblast invasion^4,5^. Impaired decidualization of endometrial stroma can lead to implantation failure, miscarriage, and unexplained infertility^1,6^. During decidualization, hESCs dramatically change their fibroblast-like morphology into the epithelial-like state with the dynamic rearrangement of cytoplasmic actin^7^. Interestingly, this cytoskeletal actin dynamics not only morphologically, but also functionally regulate decidualization^8,9^. Therefore, many studies have focused on the roles of cytoplasmic actin in decidualization^10,11^.

Recently, it has become clear that actin exists in the nucleus as well and plays crucial roles in the regulation of transcription^12,13^. Actin is found in all kinds of RNA polymerase complexes and in a number of chromatin remodeling complexes^14–17^. As is well known for cytoplasmic actin, nuclear actin shows dynamic changes of its polymerized state between monomeric globular (G-) and polymerized filamentous (F-) actin forms^18,19^. The equilibrium between G- and F-actin affects crucial nuclear events such as DNA damage response^20,21^. It was reported that nuclear actin assembly is induced upon external and internal stimuli^13,22^ and is involved in the regulation of transcription, chromatin remodeling, DNA-damage repair, and DNA replication^23–25^. Recent reports suggest that nuclear actin assembly is important for mouse embryonic development and cellular differentiation^12,26–29^. However, it is still unclear whether nuclear actin assembly is involved in the regulation of physiological functions in human organs.

ESCs derived from human endometrium can recapitulate the decidualization process upon supplementation of dibutyryl cAMP, in which cytoskeletal actin reorganization and morphological transformation are observed^7^. Using this well-established decidualization model, we examined nuclear actin dynamics in primary cultured hESCs. For visualizing nuclear actin dynamics, live cell imaging was performed by taking advantage of the nuclear actin chromobody-GFP (nAC-GFP) probe^30^ and we found that nuclear actin is specifically assembled in hESCs undergoing decidualization. The forced disassembly of nuclear actin prevented efficient decidualization of hESCs and resulted in impaired downregulation of a specific set of genes that are normally repressed during decidualization. Especially, genes related to cellular proliferation were kept activated after nuclear actin disassembly, and abnormal growth of hESCs was observed. Mechanistically, we showed that a pioneer transcription factor CCAAT/enhancer-binding protein beta (C/EBPβ) was related to the downregulation of exportin 6 (XPO6) protein, a specific exporter for nuclear actin, during decidualization, leading to the accumulation of nuclear actin. These studies demonstrate that nuclear actin assembly is an important part of establishing the decidualized state.

## Results

### Nuclear actin assembly in human endometrial stromal cells undergoing decidualization

**Fig. 1:**
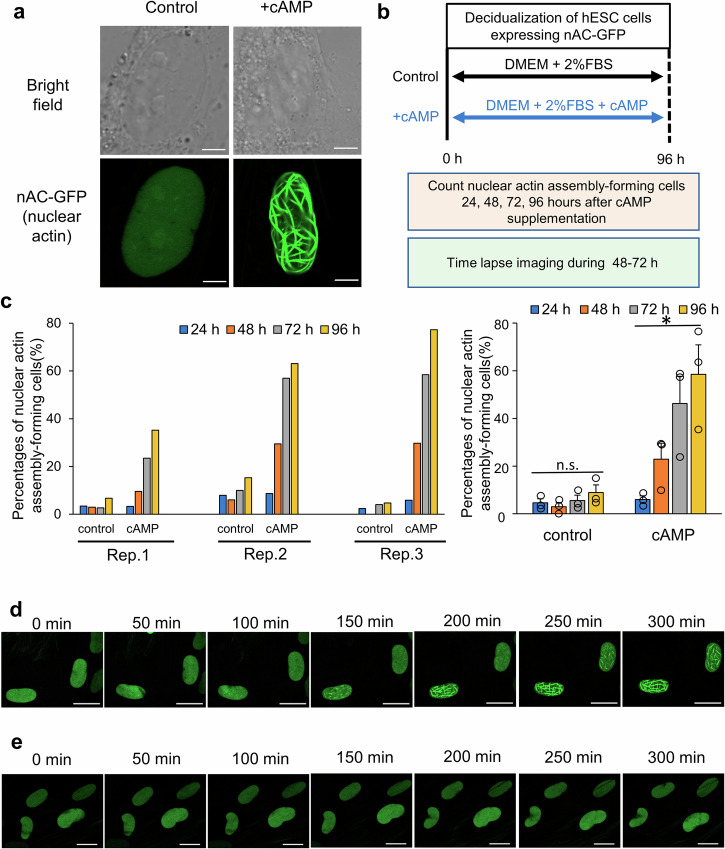
Nuclear actin is assembled after cAMP-mediated decidualization of hESCs. **a** A representative image of a hESC that showed the network of nuclear actin assembly after transient expression of nuclear actin chromobody (nAC)-GFP that probes nuclear actin. hESCs were treated with cAMP for 96 h. The control represents hESCs that were not treated with cAMP as shown in Supplementary Fig. 1a. Scale bars, 5 μm. **b** A schematic diagram for observing the formation of nuclear actin assembly during decidualization of hESCs stably expressing nAC-GFP. **c** Percentages of cells that showed nuclear actin assembly. Cells were established from three different patients (Rep. 1–3) and were treated with or without cAMP for 96 h. Cells that showed nuclear actin assembly and network of nuclear actin assembly (Supplementary Fig. 2) were both counted as being positive for the formation of nuclear actin assembly. Hours after cAMP supplementation are indicated. Left, data of each case. Right, mean ± SE of three cases. Each data point is indicated as a dot. *P < 0.05 (Tukey-Kramer test). n.s. represents not significant. **d, e** Representative images that capture the formation of nuclear actin assembly during decidualization. Images were taken from 57 h after cAMP supplementation (**d**). As a control, no cAMP-supplemented control cells cultured in DMEM with 2% FBS were also captured by confocal microscopy under the same setting (**e**). Scale bars, 20 μm.

We investigated nuclear actin dynamics during decidualization of hESCs. For live cell imaging of nuclear actin dynamics, hESCs were treated with or without cAMP, a stimulus for decidualization, for 96 h^31^, and nAC-GFP^30^ was transfected for nuclear actin visualization at 72 h (Supplementary Fig. 1a). Actin filaments were assembled in nuclei of cAMP-treated cells (Fig. 1a) with the induction of insulin-like growth factor binding protein 1 (IGFBP1) and prolactin (PRL) mRNA expression (Supplementary Fig. 1b), specific markers for decidualization^7^. The assembled nuclear actin was interconnected each other and formed the networked structure (Fig. 1a). To note, expression of nAC-GFP did not affect the nuclear actin levels in hESCs (Supplementary Fig. 1c) and, moreover, cytoplasmic actin levels were not altered between decidualized ESCs expressing nAC-GFP and those expressing control mCherry protein (Supplementary Fig. 1d), suggesting that expression of the nAC-GFP probe itself does not affect actin amounts in hESCs. We next established hESCs derived from three women that stably expressed nAC-GFP, and these cells were subjected to cAMP treatment and subsequent live cell imaging (Fig. 1b). Striking increases in the percentages of cells that showed nuclear actin assembly were observed after supplementation of cAMP (Fig. 1c and Supplementary Fig. 1e; 6.0 ± 1.6% at 24 h to 58.5 ± 12.3% at 96 h as mean ± SE of Rep. 1–3 of cAMP shown in Fig. 1c, *P* < 0.05 by Tukey test). Proportions of cells that formed nuclear actin assembly were variable among women (Fig. 1c and Supplementary Fig. 2). However, all responded to cAMP and significant increases were observed especially in the proportions of cells exhibiting the network of nuclear actin assembly compared with control (cAMP-untreated) cells (Supplementary Fig. 2, *P* < 0.01).

To observe endogenous F-actin dynamics during decidualization, wild type hESCs were treated with or without cAMP for 96 h. The morphological change to the epithelial-like state was observed under this condition (Supplementary Fig. 3a, +cAMP). Phalloidin staining also showed the epithelial-like shape of cAMP-treated cells while cells without cAMP remained as the fibroblast-like morphology (Supplementary Fig. 3b). The obvious changes in the cytoplasmic phalloidin-stainable F-actin amounts were not observed, while nuclear F-actin is more apparent in cAMP-treated hESCs than non-treated cells (Supplementary Fig. 3c). The network of nuclear actin assembly as detected with nAC-GFP was not observed with phalloidin staining. Nevertheless, these results suggest that nuclear actin assembly is enhanced in decidualized cells.

We then captured the assembling process of nuclear actin by time lapse imaging using hESCs stably expressing nAC-GFP during 48 h to 72 h after the cAMP stimulation. It showed that the nuclear actin was assembled within hours (Fig. 1d and Supplementary Movie 1, 2), while the same nAC-GFP-expressing cells without the cAMP stimulation did not show nuclear actin assembly (Fig. 1e). These results demonstrate that nuclear actin assembly is observed specifically in the process of decidualization in hESCs.

### Nuclear actin in decidualized hESCs is disassembled after withdrawing cAMP

**Fig. 2:**
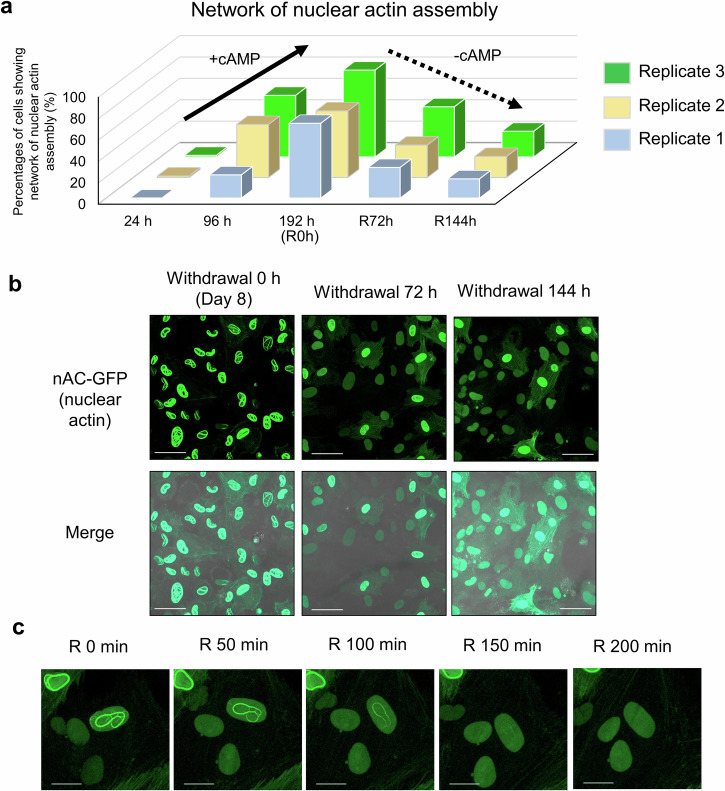
Nuclear actin is disassembled by reversing the decidualized state of hESCs. **a** Percentages of cells that showed the network of nuclear actin assembly. Cells were established from three different patients (Rep. 1–3). Cells that showed the network of nuclear actin assembly (Supplementary Fig. 2) were counted. Hours after cAMP supplementation are indicated. After 192 h of culture with cAMP, cells were cultured without cAMP for recovery (R0h). Cells were counted at 72 h (R72h) and 144 h (R144h) after the removal of cAMP. Data of 24 h and 96 h are same as Fig. 1c. **b** Representative confocal images of decidualized hESC cells expressing nAC-GFP after withdrawal of cAMP. Times after cAMP withdrawal are indicated above (Recovery). Merged images of GFP channels and DIC are shown (Merge). Scale bars, 50 μm. **c** Representative images that follow a process of nuclear actin disassembly after withdrawal of cAMP. Images were taken from 73 h after cAMP withdrawal. Recovery times are shown. Scale bars, 20 μm.

It is known that decidualization is a reversible process and the removal of cAMP from the culture medium is sufficient to convert the decidualized hESCs to the original non-decidualized state^32,33^. We then asked if the nuclear actin filaments detected in the cAMP-treated hESCs is disassembled by removing cAMP. hESCs were further treated with cAMP until 8 days (192 h). Approximately 71.1 ± 5.5% (Mean ± SE of Rep. 1–3) of cells showed the network of nuclear actin assembly (Fig. 2a), and then cAMP was withdrawn from the culture medium. The sharp decrease in the percentages of cells showing nuclear actin assembly was observed (Fig. 2a, b; 20.5 ± 1.8% at R144h as mean ± SE of Rep. 1–3). The disassembly process of nuclear actin was observed within hours (Fig. 2c and Supplementary Movie 3), being similar to the case of nuclear actin assembly (Fig. 1d and Supplementary Movie 1). These results suggest that the assembly of nuclear actin in hESCs is a reversible process and is correlated with the decidualized state of the cells.

### Nuclear actin assembly is needed for the decidualization of hESCs

**Fig. 3:**
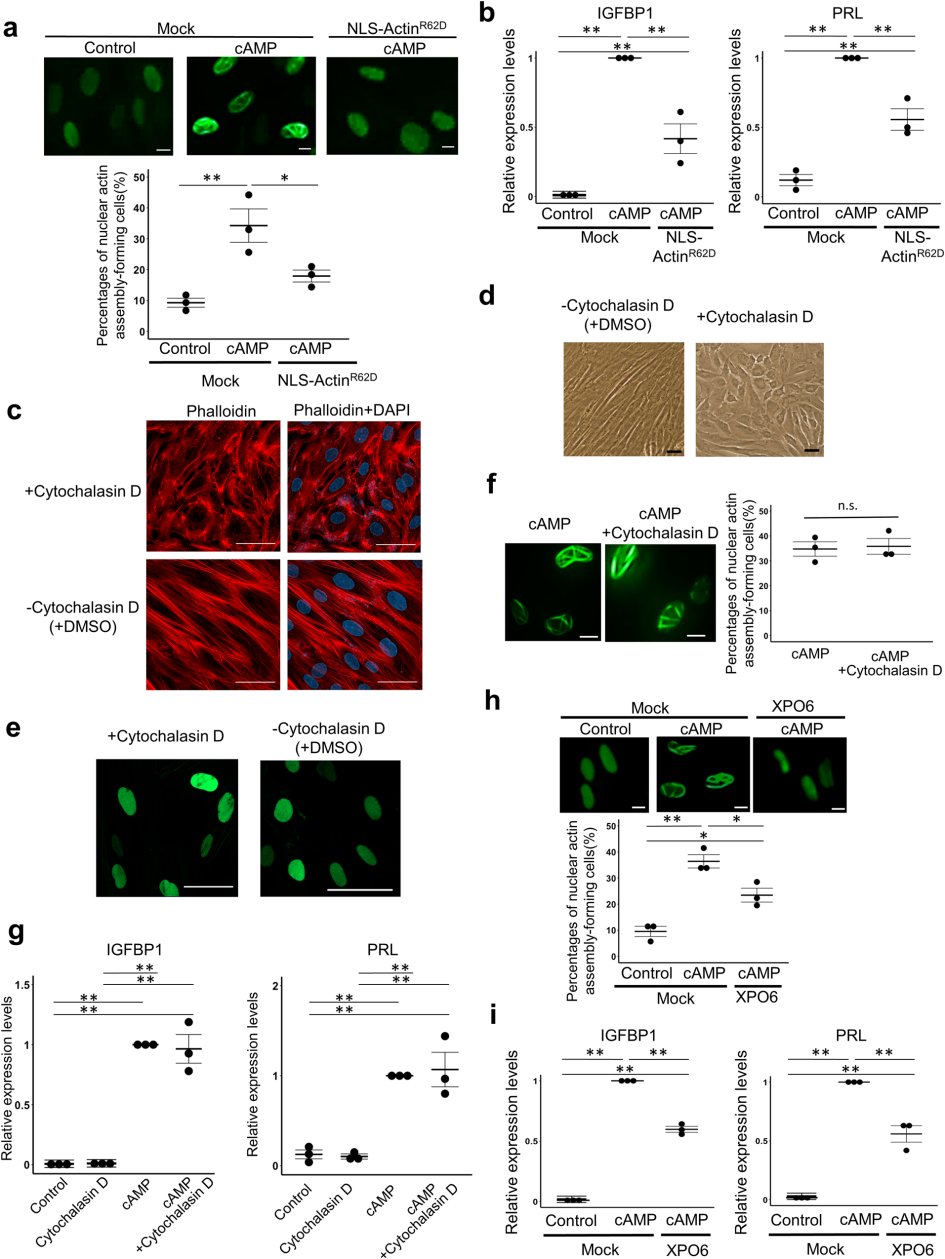
Nuclear actin assembly is required for decidualization of hESCs. **a** The effect of overexpressing NLS-Actin^R62D^ on the formation of nuclear actin assembly during decidualization. hESCs stably expressing nAC-GFP were overexpressed with mCherry (as a mock control) or NLS-Actin^R62D^, and were treated with or without cAMP for 96 h. Representative images and percentages of cells that showed nuclear actin assembly are indicated. Mean ± SE of three independent experiments. Each data point is indicated as a dot. **P < 0.01; *P < 0.05 (Tukey-Kramer test). Scale bars, 10 μm. **b** RT-qPCR analyses of decidualization markers (IGFBP1 and PRL) after overexpressing NLS-Actin^R62D^. hESCs were overexpressed with mCherry (as a mock control) or NLS-Actin^R62D^, and were treated with or without cAMP for 96 h. Relative expression levels to cAMP-treated mock hESCs are shown. Mean ± SE of three independent experiments. Each data point is indicated as a dot. **P < 0.01 (Tukey-Kramer test). **c** Endogenous F-actin stained with phalloidin in hESCs treated with Cytochalasin D or DMSO (vehicle control). DNA was visualized by DAPI. Scale bars, 50 μm. **d** A bright filed image of hESCs after 4 days of Cytochalasin D treatment. As a control, hESCs were cultured in DMEM with 2% FBS with DMSO. Scale bar, 50 μm. **e** Representative images of hESCs expressing nAC-GFP treated with or without Cytochalasin D for 4 days. Scale bars, 50 μm. **f** Representative images of cAMP-treated hESCs expressing nAC-GFP, co-cultured with or without Cytochalasin D for 4 days. Scale bars, 10 μm. Percentages of cells that showed nuclear actin assembly are indicated. Mean ± SE of three independent experiments. Each data point is indicated as a dot. n.s. represents not significant (Student’s *t* test). **g** RT-qPCR analyses of decidualization markers (IGFBP1 and PRL) in cAMP-treated hESCs co-cultured with or without Cytochalasin D for 4 days. Relative expression levels to cAMP-treated hESCs are shown. Mean ± SE of three independent experiments. Each data point is indicated as a dot. **P < 0.01 (Tukey-Kramer test). **h** The effect of overexpressing XPO6 on the formation of nuclear actin assembly during decidualization. hESCs stably expressing nAC-GFP were overexpressed with mCherry (as a mock control) or XPO6, and were treated with or without cAMP for 96 h. Representative images and percentages of cells that showed nuclear actin assembly are indicated. Mean ± SE of three independent experiments. Each data point is indicated as a dot. **P < 0.01; *P < 0.05 (Tukey-Kramer test). Scale bars, 10 μm. **i** RT-qPCR analyses of decidualization markers (IGFBP1 and PRL) after overexpressing XPO6 in cAMP-treated hESCs. hESCs were overexpressed with mCherry (as a mock control) or XPO6, and were treated with or without cAMP for 96 h. Relative expression levels to cAMP-treated mock hESCs are shown. Mean ± SE of three independent experiments. Each data point is indicated as a dot. **P < 0.01 (Tukey-Kramer test).

We next asked if the nuclear actin assembly in hESCs is important for decidualization. The nuclear actin polymerization can be specifically inhibited by the overexpression of an actin mutant Actin^R62D^ in the nucleus that does not polymerize^34^. Overexpression of Actin^R62D^ tagged with the nuclear localization signal (NLS-Actin^R62D^) significantly reduced the number of cells exhibiting the nuclear actin assembly induced by cAMP stimulation (Fig. 3a, *P* < 0.05). In accordance with the reduction of nuclear actin assembly, the full upregulation of decidualization markers (IGFBP1 and PRL) by cAMP was impaired by the overexpression of NLS-Actin^R62D^ (Fig. 3b, *P* < 0.01). It is still possible that NLS-Actin^R62D^ was exported to the cytoplasm and disturbed cytoplasmic actin assembly, leading to the defects in decidualization. We therefore asked the contribution of actin assembly in the cytoplasm to decidualization by treating decidualizing cells with Cytochalasin D, an actin depolymerizing reagent, at a mild concentration. The mild concentration of Cytochalasin D induces depolymerization of cytoplasmic actin, but not that of nuclear actin^35^. We explored an appropriate concentration of Cytochalasin D, which did not impair cell survival, but simultaneously depolymerized cytoplasmic actin in hESC. Then, the treatment of hESCs with 0.05 μM of Cytochalasin D did not compromise the viability of hESCs and induced rearrangement of actin cytoskeleton within 48 h of treatment (Fig. 3c). Interestingly, the epithelial-like morphology was established by Cytochalasin D without cAMP supplementation (Fig. 3d), suggesting that the morphological change from the fibroblast-like to epithelial-like shape is accompanied by cytoplasmic actin rearrangement in good agreement with the previous finding^9,36^. However, the Cytochalasin D treatment itself was not sufficient to trigger nuclear actin assembly at least with this concentration (Fig. 3e). We next investigated the effect of the Cytochalasin D treatment on cAMP-mediated decidualization of hESCs. The treatment of hESCs with 0.05 μM Cytochalasin D neither inhibited cAMP-induced nuclear actin assembly (Fig. 3f) nor decidualization as judged by marker expression (Fig. 3g). Taken together, actin dynamics in the cytoplasm play a role in the morphological change during decidualization and those in the nucleus may be important for establishing the gene expression profile specific for decidualized cells. Furthermore, the inhibitory effect of NLS-Actin^R62D^ overexpression on decidualization is unlikely through the depolymerization of cytoplasmic actin since the forced depolymerization of cytoplasmic actin with Cytochalasin D did not interfere with decidualization.

We then examined the effect of XPO6 overexpression on the expression of decidualization markers. Overexpression of XPO6 is recognized as another reliable method to inhibit the nuclear actin polymerization by specifically exporting nuclear actin and thus reducing nuclear pools^37^. Indeed, the overexpression of XPO6 reduced the number of cells showing the nuclear actin assembly (Fig. 3h, *P* < 0.05) and impaired the full upregulation of IGFBP1 and PRL in cAMP-treated hESCs (Fig. 3i, *P* < 0.01). Taken together, nuclear actin assembly is required for accomplishing decidualization.

### Nuclear actin assembly suppresses cell proliferation during decidualization

**Fig. 4:**
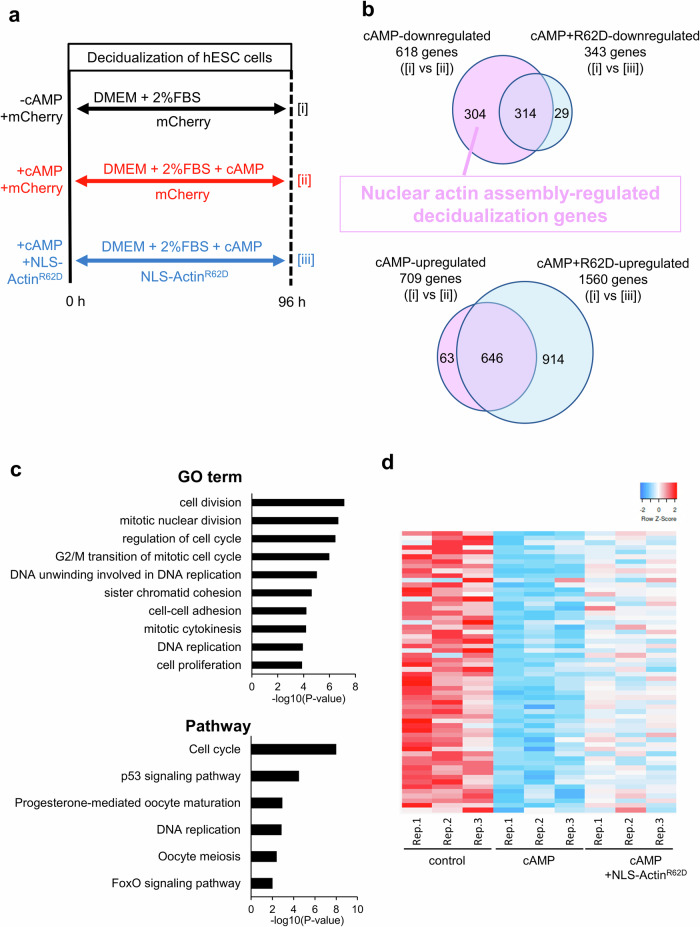
Incomplete downregulation of decidualization-related genes after forced disassembly of nuclear actin in hESCs undergoing decidualization, as revealed by RNA-seq analyses. **a** A schematic diagram for RNA-seq analyses. Three different types of samples were collected (i–iii). **b** Ven diagrams show the number of total and overlapping genes. Up- or down-regulated genes were identified (*p* adj < 0.05) by comparing samples i vs ii or i vs iii, as shown in Fig. 4a. Genes that are downregulated by cAMP treatment, but are not downregulated by overexpressing NLS-Actin^R62D^ are termed as “nuclear actin assembly-regulated decidualization genes”. **c** GO and pathway analyses of nuclear actin assembly-regulated decidualization genes, identified in Fig. 4b. **d** A heatmap shows expression levels of 60 genes related to cellular proliferation, selected from nuclear actin assembly-regulated decidualization genes.

**Fig. 5:**
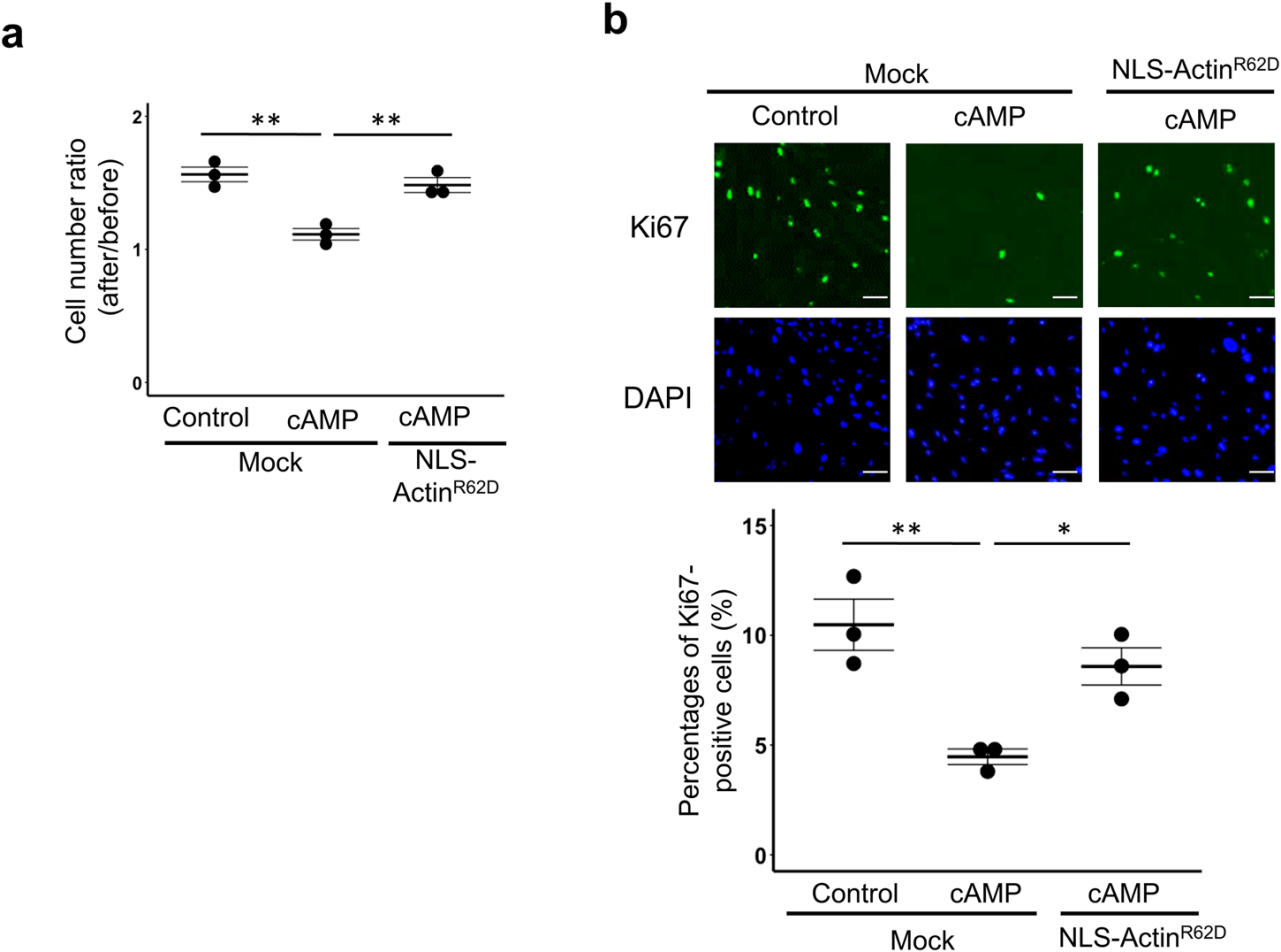
Cell cycle arrest of hESCs after cAMP stimulation is inhibited by forced disassembly of nuclear actin. **a** hESCs were overexpressed with mCherry (as a mock control) or NLS-Actin^R62D^. Cell numbers were counted before and after 96 h of incubation with or without cAMP, and the ratios were compared. NLS-Actin^R62D^ overexpression increased cell numbers that were normally unchanged after cAMP stimulation. Mean ± SE of three independent experiments. Each data point is indicated as a dot. **P < 0.01 (Tukey-Kramer test). **b** hESCs were overexpressed with mCherry (as a mock control) or NLS-Actin^R62D^. Percentages of Ki67-positive cells were counted after 96 h of incubation with or without cAMP. NLS-Actin^R62D^ overexpression increased Ki67-positive proliferating cells, when compared to the mock control with cAMP. Mean ± SE of three independent experiments. Each data point is indicated as a dot. **P < 0.01; *P < 0.05 (Tukey-Kramer test). Scale bars, 100 μm.

In order to investigate how the nuclear actin assembly is involved in decidualization, we performed RNA-seq analysis of hESCs derived from three different women, in which the inhibitory effect of NLS-Actin^R62D^ overexpression on decidualization was confirmed (Supplementary Fig. 4). We prepared three treatments for the comparison: control hESCs (cAMP-untreated cells with overexpression of control mCherry protein) (Fig. 4a, [i]), cAMP-treated hESCs with overexpression of control mCherry protein or NLS-Actin^R62D^ (Fig. 4a, [ii] and [iii], respectively). By comparing non-decidualized and decidualized hESCs ([i] vs [ii]), we have identified 618 downregulated and 709 upregulated genes (*p* adj < 0.05). Among the 618 genes that should be repressed in the course of decidualization, the downregulation of 304 genes was not observed when cells were overexpressed NLS-Actin^R62D^ (Fig. 4b). These 304 genes were defined as “nuclear actin assembly-regulated decidualization genes” (Supplementary Table 1). Gene ontology (GO) and KEGG pathway analyses showed that they were related to the regulation of cell proliferation (Fig. 4c, Supplementary Table 2). Expression levels of 60 genes associated with cell proliferation were shown in the heat map (Fig. 4d), suggesting that their expression levels were downregulated by cAMP and the decreases in expression levels were partially suppressed by the overexpression of NLS-Actin^R62D^. On the other hand, most of the 709 genes that should be upregulated during decidualization (Fig. 4b) were properly upregulated after overexpression of NLS-Actin^R62D^ and only 63 genes were not activated by NLS-Actin^R62D^ overexpression (Fig. 4b). GO analysis identified few terms in these 63 genes (Supplementary Table 3). Therefore, disassembly of nuclear actin resulted in impaired downregulation, rather than upregulation, of genes involved in decidualization. In particular, its target genes are involved in cell proliferation. It has been shown that, after cAMP stimulation, ESCs have to exit the cell cycle for accomplishing their differentiation process towards the decidualized state^38^. Therefore, we asked if the nuclear actin assembly is involved in the suppression of cell proliferation during decidualization. The cell number of cAMP-treated cells was significantly lower than that of cAMP-untreated cells, showing that decidualization stimuli inhibit cell proliferation (Fig. 5a). Overexpression of NLS-Actin^R62D^ suppressed the cAMP-induced decrease in cell number (Fig. 5a). Similar results were observed in Ki67 staining, a marker for proliferating cells (Fig. 5b). These results indicate that nuclear actin assembly is involved in the suppression of cell proliferation during decidualization through the regulation of proliferation-related genes.

### Identification of an upstream factor regulating nuclear actin assembly

**Fig. 6:**
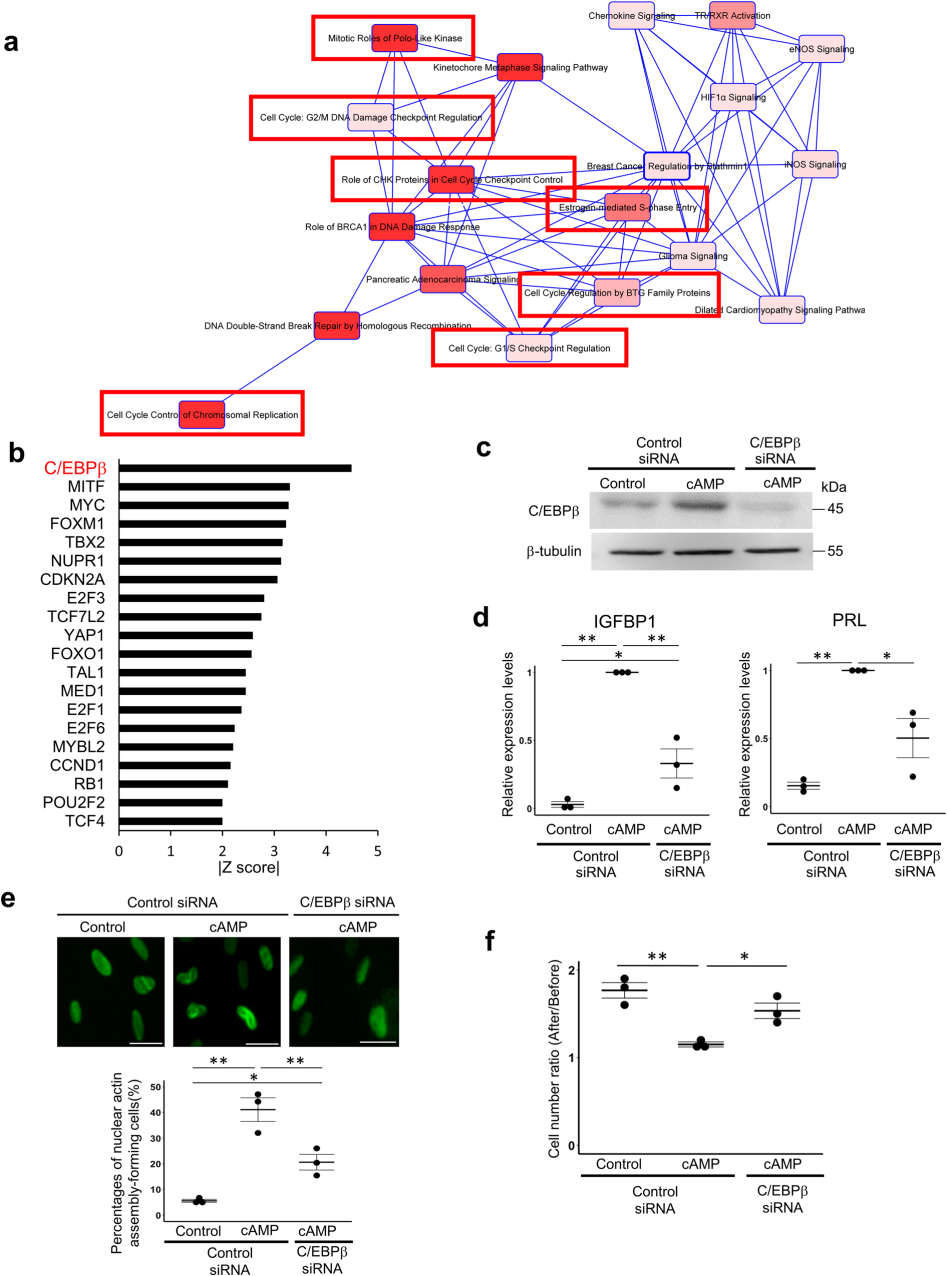
C/EBPβ contributes to nuclear actin assembly in decidualized hESCs. **a** Canonical pathways predicted by IPA using nuclear actin assembly-regulated decidualization genes. Many cell cycle-related terms are found (red boxes). Terms with strong significance are indicated as intense red. **b** Upstream regulators for nuclear actin assembly-regulated decidualization genes are predicated by IPA. Among them, top 20 transcription regulators based on the absolute values of z-score are shown. **c** Representative images of western blot analyses of cAMP-treated hESCs with or without siRNA-mediated knockdown of C/EBPβ. hESCs were transfected with siRNA against C/EBPβ or control siRNA, and were treated with or without cAMP for 96 h. Control represents hESCs not treated with cAMP. Antibodies against C/EBPβ and β-tubulin are used. **d** RT-qPCR analyses of decidualization markers (IGFBP1 and PRL) in cAMP-treated hESCs transfected with siRNA. Relative expression levels to the cAMP-treated hESCs transfected with control siRNA are shown. Mean ± SE of three independent experiments. Each data point is indicated as a dot. **P < 0.01; *P < 0.05 (Tukey-Kramer test). **e** The effect of C/EBPβ knockdown on the formation of nuclear actin assembly during decidualization. Representative images and proportions of cells that showed nuclear actin assembly are indicated. Mean ± SE of three independent experiments. Each data point is indicated as a dot. **P < 0.01; *P < 0.05 (Tukey-Kramer test). Scale bars, 50 μm. **f** The effect of C/EBPβ knockdown on cell cycle arrest during decidualization. Cell numbers were counted before and after 96 h of incubation with or without cAMP, and the ratios were compared. Mean ± SE of three independent experiments. Each data point is indicated as a dot. **P < 0.01; *P < 0.05 (Tukey-Kramer test).

To gain mechanistic insight into nuclear actin-regulated gene expression, we sought to identify co-factors that are involved in the downregulation of decidualization-related genes by nuclear actin assembly. We performed Ingenuity® Pathway Analysis (IPA) to identify the molecular network and co-factors for nuclear actin assembly-related gene regulation. The canonical pathway analysis identified many terms related to cell cycle regulation, suggesting that the nuclear actin assembly-regulated decidualization genes are indeed closely related to cell cycle progression (Fig. 6a; marked with red boxes and Supplementary Fig. 5a). IPA further identified candidate upstream regulators for nuclear actin-regulated genes (*p* < 0.01, Supplementary Table 4), among which C/EBPβ is top-listed according to the activation Z-score (Fig. 6b). C/EBPβ was predicted to be an upstream factor for cell proliferation-associated proteins including TP53 and Rb (Supplementary Fig. 5b). We previously reported that C/EBPβ is an essential transcription factor for decidualization and its expression increases upon cAMP stimulation^39–43^. Therefore, we examined the effect of C/EBPβ knockdown on nuclear actin assembly in decidualizing hESCs. C/EBPβ knockdown successfully suppressed its protein expression during decidualization (Fig. 6c) and simultaneously downregulation of decidualization markers was observed (Fig. 6d). Importantly, cells exhibiting nuclear actin assembly were significantly reduced by C/EBPβ knockdown (Fig. 6e). Furthermore, the number of cells were significantly more in C/EBPβ-knockdown cells than cAMP-treated decidualized cells (Fig. 6f), suggesting that C/EBPβ knockdown impaired nuclear actin assembly and cell cycle arrest. To examine the causative relationship between C/EBPβ and nuclear actin assembly, the effect of NLS-Actin^R62D^ overexpression on C/EBPβ expression was examined. NLS-Actin^R62D^ overexpression did not affect cAMP-induced C/EBPβ upregulation at the mRNA and protein levels (Supplementary Fig. 6a, b). These results indicate that C/EBPβ serves as an upstream factor regulating nuclear actin assembly.

### Increase of nuclear actin levels is associated with the formation of nuclear actin filaments

**Fig. 7:**
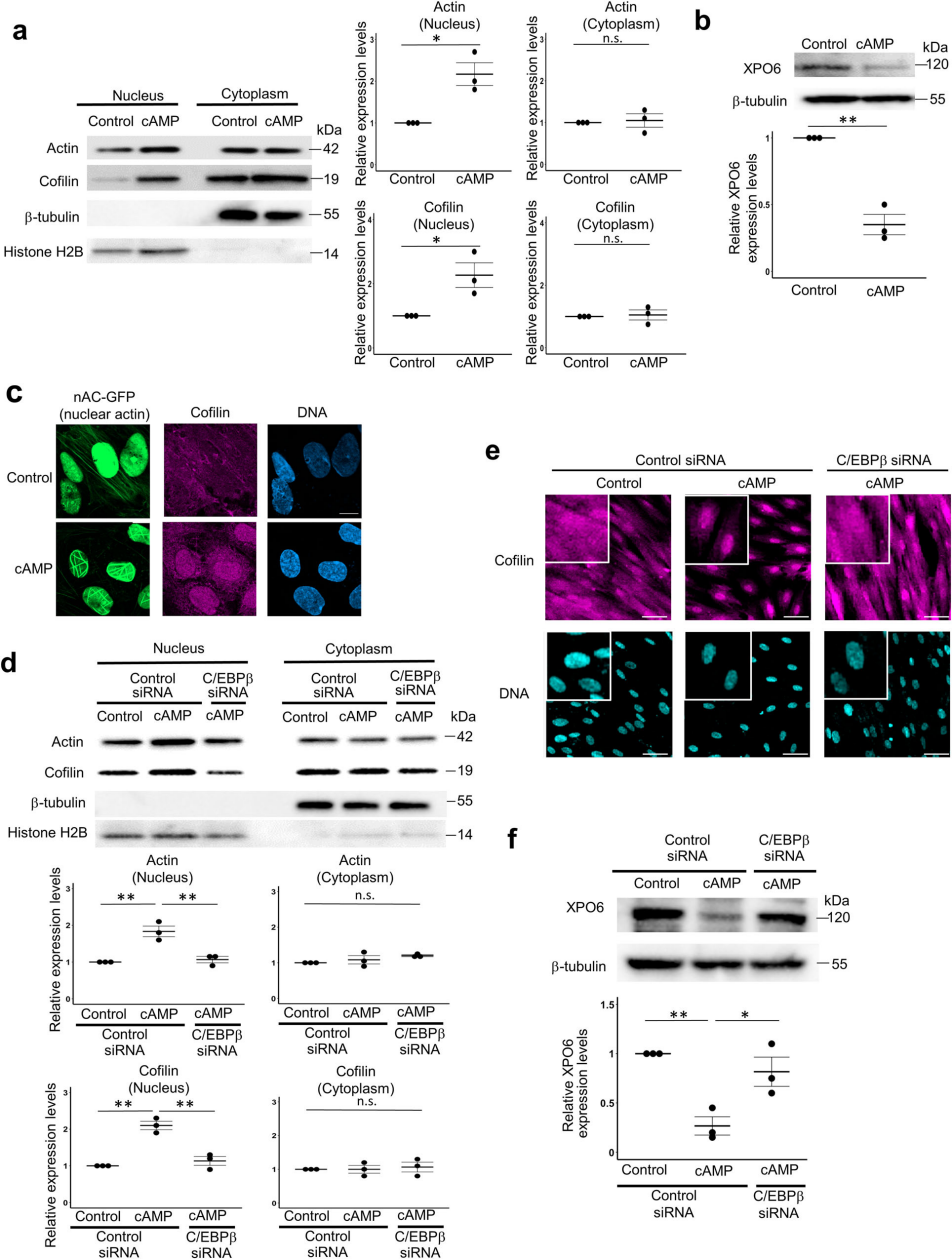
The increase of nuclear actin levels by XPO6 downregulation is associated with nuclear actin assembly during decidualization. **a** Representative images of western blot analyses of isolated hESC nuclei and cytoplasm against β-actin, cofilin, β-tubulin, and histone H2B. hESCs were treated with or without cAMP (control) for 48 h. Band intensities of actin and cofilin were quantified and the relative intensities of cAMP-treated samples to the control are shown in the graph. Mean ± SE. Each data point is indicated as a dot. *P < 0.05 (Student’s *t* test). n.s. represents not significant. Three independent experiments were repeated. **b** Representative images of western blot analyses of whole hESC lysates against XPO6 and β-tubulin. hESCs were treated with or without cAMP (control) for 96 h. Band intensities were quantified and the relative intensities of cAMP-treated samples to the control are shown in the graph. Mean ± SE. Each data point is indicated as a dot. **P < 0.05 (Student’s *t* test). Three independent experiments were repeated. **c** Accumulation of cofilin in nuclei after induction of cAMP-stimulated decidualization of hESCs. Representative immunofluorescence images of hESCs stably expressing nAC-GFP treated with cAMP for 96 h. As a control, hESCs without cAMP addition were examined (control). DNA was stained with DAPI. Scale bar, 10 μm. **d** The effect of C/EBPβ knockdown on the increase of nuclear actin and cofilin during decidualization. Representative images of western blot analyses of isolated hESCs nuclei against β-actin, cofilin, β-tubulin, and histone H2B are shown. Band intensities were quantified and the relative intensities to no-treated hESCs transfected with control siRNA are shown (control). Mean ± SE of three independent experiments. Each data point is indicated as a dot. **P < 0.01 (Tukey-Kramer test). n.s. represents not significant. **e** The effect of C/EBPβ knockdown on the nuclear accumulation of cofilin after induction of cAMP-stimulated decidualization of hESCs. hESCs were transfected with siRNA against C/EBPβ or control siRNA, and were treated with or without cAMP. Representative immunofluorescence images are shown. As a control, hESCs without cAMP addition were examined (control). DNA was stained with DAPI. Scale bar, 50 μm. **f** The effect of C/EBPβ knockdown on XPO6 expression during decidualization. Representative images of western blot analyses of isolated hESCs nuclei against XPO6 and β-tubulin are shown. Band intensities were quantified and the relative intensities to control cAMP-untreated hESCs transfected with control siRNA (control) are shown. Mean ± SE of three independent experiments. Each data point is indicated as a dot. **P < 0.01; *P < 0.05 (Tukey-Kramer test).

We then investigated the mechanism of nuclear actin assembly during decidualization. A recent report showed that the increase of nuclear actin levels is associated with nuclear actin assembly^35^. Therefore, we examined whether the total level of nuclear actin increases along with decidualization by western blots of isolated hESC nuclei. The nuclear actin level increased by cAMP stimulation whereas the cytoplasmic actin level did not change (Fig. 7a). The nuclear actin level is determined by the regulation of import and export of actin^44,45^, for which XPO6 is known to show developmentally regulated expression dynamics^46^, We therefore examined whether the expression of XPO6 was altered in decidualizing hESCs. After cAMP treatment, the protein level of XPO6 significantly decreased (Fig. 7b). These results indicate that a decidualization stimulus increases nuclear actin levels through the decrease of XPO6, which results in the formation of nuclear actin assembly in hESCs. When actin translocates from the cytoplasm into the nucleus, it is imported together with the small actin-binding protein, cofilin^44^. After stimulation with cAMP, the increase of nuclear cofilin level and nuclear localization of cofilin were observed in hESCs (Fig. 7a, c), suggesting that actin together with cofilin are accumulated in nuclei during decidualization, in good agreement with the previous finding^9^. Because C/EBPβ was identified as an upstream factor regulating nuclear actin assembly (Fig. 6e), we examined the involvement of C/EBPβ in the regulation of nuclear actin levels. The increases of nuclear levels of actin and cofilin by cAMP were suppressed by C/EBPβ knockdown (Fig. 7d) with the inhibition of cAMP-induced nuclear localization of cofilin (Fig. 7e). C/EBPβ knockdown also suppressed the decrease of XPO6 levels by cAMP (Fig. 7f). These results indicate that C/EBPβ downregulates XPO6 expression and thus increase the total nuclear actin amount, culminating in nuclear actin assembly.

## Discussion

Decidualization of hESCs is characterized by rapid and dynamic changes in the cellular state, among which the prominent reorganization of subcellular structures has been observed^47–49^. In particular, the F‐actin cytoskeleton network of non‐decidualized hESCs is re-distributed to the periphery of the cells during decidualization^8,10^, along with the morphological change of fibroblast-like hESCs to the epithelial-like state^7^. These cytoskeletal actin dynamics functionally and morphologically affect endometrial decidualization^8,9^. In this study, we discovered that remarkable re-distribution of actin is also observed in the nucleus of decidualizing hESCs and that the newly assembled nuclear actin regulates decidualization through the inhibition of cell proliferation. We thus reveal that the reorganization of the actin nucleoskeleton is also a part of human decidualization.

One striking observation in our study is that nuclear actin is assembled after the addition of cAMP, a decidualization stimulus, and disassembled after its removal. It should be noted that decidualization is a reversible process because withdrawal of decidualization stimuli converts decidualized ESCs to a non-decidualized state^32,33^. Taken together, the reversible nuclear actin assembly is strongly associated with the decidualization status. The efficiency of nuclear actin assembly varied among patients (Fig. 1c). It is known that hESCs derived from patients with recurrent implantation failures show defects in decidualization^50,51^, suggesting variable efficiencies of decidualization depending on individuals. The close association of nuclear actin assembly and the decidualized state of hESCs led us to speculate a functional role of nuclear actin assembly in decidualization. RNA-seq analyses revealed that genes related to cellular proliferation are not properly downregulated when nuclear actin assembly is impaired. It has been shown that hESCs have to exit the cell cycle for accomplishing their differentiation process towards the decidualized state^38^. A number of genes associated with cell proliferation are downregulated during decidualization^52,53^. Nuclear actin assembly is involved in the downregulation of such genes to suppress cellular proliferation for decidualization.

Previous reports have found that nuclear F-actin is transiently formed in early G1 phase (disappear within a few hours)^18^ and required for the initiation of DNA replication^54^. In contract, our observed nuclear actin assembly is stably formed more than days as long as decidualization stimuli are supplemented. Furthermore, nuclear actin assembled in decidualized ESCs showed thicker structures (Supplementary Movie 2) than those observed in previous studies^18,30,54^. Taken these results together, nuclear actin assembled in decidualized hESCs clearly show different characteristics from the early G1 F-actin. This idea is further supported by the functional difference between the transiently formed nuclear F-actin and our observed nuclear actin assembled in hESCs. The transiently formed nuclear F-actin including the early G1 F-actin is involved in chromatin decondensation and gene activation^18^, while our observed nuclear actin is important for repressing cell proliferation genes (Fig. 4). Further studies are needed to clarify the molecular mechanisms underlying nuclear actin assembly-related gene repression during decidualization. Recent studies show that nuclear actin polymerization is responsible for re-organization of the genome following DNA damage^55^ and nuclear β-actin profoundly affects the genome organization^56^ and enhancer activities through influencing H3K27 acetylation^57^. Therefore, it is interesting to ask how actin-based changes of the nucleoskeleton structure affect the global epigenome state for gene repression during decidualization^52,53^. Especially, we have previously shown that genome-wide re-distribution of H3K27 acetylation by C/EBPβ is key to decidualization^52^, and therefore nuclear actin assembly might affect deposition of H3K27 acetylation during decidualization. Genome-wide analyses of H3K27 acetylation in hESCs with or without a disturbance in nuclear actin assembly will provide mechanistic insight into how expression of specific genes is affected by nuclear actin assembly.

C/EBPβ was identified as an upstream factor regulating nuclear actin assembly. Decidualization stimuli activate a number of transcription factors in hESCs^7,41–43,52,53^. C/EBPβ is one of them and is a critical transcription factor for decidualization^40–42,52,58,59^. We previously reported that C/EBPβ regulates about half of genes whose expression is altered during decidualization^52^. It also works as a pioneer factor initiating chromatin remodeling of the promoters and enhancers of decidualization-related genes^41,42,52^. In this study, we showed that knockdown of C/EBPβ impaired nuclear actin assembly with the disruption of cell cycle arrest. It is still possible that knockdown of C/EBPβ compromised the induction of hESCs to the decidualized state, and as a result nuclear actin assembly was impaired. Future studies should focus on the hierarchical molecular events that are induced between C/EBPβ activation and nuclear actin assembly for the comprehensive understanding of human decidualization.

An intriguing question would be how the assembly of nuclear actin is dynamically regulated. A recent report showed that the amount of nuclear actin dictates the formation of nuclear actin assembly ^35^. Considering that the total actin level is increased in the nucleus of hESCs upon supplementation of the decidualization stimulus (Fig. 7a), the increased nuclear actin results in the induction of nuclear actin assembly. In addition, nuclear localization of cofilin (Fig. 7c) is reminiscent of the increase of nuclear actin amounts because cofilin forms a complex with cytoplasmic actin for their import into the nucleus^44^, although it is unclear how our observed nuclear actin filaments in decidualized hESCs are stably maintained over days in the presence of a depolymerizing factor, Cofilin. After the import of cytoplasmic actin to the nucleus, the regulation of nuclear actin amount depends on the export of nuclear actin from the nucleus to cytoplasm by XPO6^60^. The XPO6-mediated control of nuclear actin amount has also been reported in normal mammary cells^61^. Because XPO6 was downregulated during decidualization (Fig. 7b), the decidualization stimulus increases nuclear actin levels by decreasing XPO6, which results in the assembly of nuclear actin. This model that the nuclear actin amounts control its assembly is fit to our observed kinetics of actin assembly/disassembly processes which start days after stimuli and require hours to complete (Figs. 1d, 2c); for example, in the course of macrophage differentiation accumulation of nuclear actin takes more than 24 h^62^ and then actin polymerization may be initiated once it reaches to a critical concentration. In addition, the treatment of hESCs with the mild concentration of Cytochalasin D resulted in depolymerization of cytoplasmic actin, but the subsequent nuclear actin assembly and decidualization were not disturbed (Fig. 3f, g). These results imply that cytoskeletal actin depolymerization might result in the increased pool of monomeric actin to be imported to nuclei for subsequent accumulation and assembly of nuclear actin. It is possible that an active nuclear import process of actin and/or another specific export mechanism of nuclear actin such as RASSF1A^63^ might also be involved in the regulation of nuclear actin amounts.

The following limitations of the current study should be addressed. Firstly, the precise molecular mechanism of C/EBPβ-mediated repression of XPO6 is unclear. Knockdown of C/EBPβ resulted in accumulation of XPO6 protein (Fig. 7f). However, *XPO6* mRNA levels were not changed after C/EBPβ knockdown and the C/EBPβ binding site was not found near the promoter of human *XPO6*. Taken together, it is unlikely that C/EBPβ directly represses *XPO6* mRNA expression, and rather C/EBPβ may regulate expression of an unknown factor that controls XPO6 translation. Secondly, the structure of nuclear actin assembled in decidualized ESCs is not well defined. The assembled actin in decidualized ESCs is different from ‘nuclear actin rods’, a larger polymer of actin^23^, and the recently reported phalloidin-negative nuclear actin filaments^64^, because the clear accumulation of cofilin onto nuclear actin filaments was not observed in our study. A new actin-visualizing tool such as the fluorescent labeling of endogenous actin might help to clarify the nuclear actin structure.

In summary, our study reveals the physiological role of nuclear actin assembly in the course of differentiation of human primary endometrial stromal cells. It suppresses cell proliferation for differentiation towards the decidualized state. Cytoskeletal reorganization has been well documented during decidualization, and our study sheds light on dramatic reorganization of the nucleoskeleton as a part of decidualization. The interplay between cytoplasmic and nuclear actin needs to be also investigated in the context of mechanotransduction as actin might work as a critical regulator for transmitting cytoskeletal cues to chromatin for accomplishing decidualization during implantation in human.

## Methods

### hESC isolation

Human endometrial tissues were obtained at hysterectomy from patients with a normal menstrual cycle, aged 40–45 years, who underwent surgery for myoma uteri or early stage cervical cancer. The patients were not on hormonal therapy at the time of surgery. Informed consent was obtained from all participating patients, and ethical approval was obtained from the Institutional Review Board of Yamaguchi University Hospital (H26-102-7). All experiments were performed in accordance with the Tenets of the Declaration of Helsinki. All ethical regulations relevant to human research participants were followed. Endometrial samples utilized for ESC isolation were histologically diagnosed as being in the late proliferative phase according to the published criteria^65^. Tissue samples were washed with Phenol Red-free DMEM containing 4 mM glutamine, 50 mg/ml streptomycin and 50 IU/ml penicillin, and minced into pieces of <1 mm^3^. ESCs were isolated as reported previously^66^. Cells were seeded at 10^5^ cells/cm^2^ in 75 cm^2^ tissue culture flasks and incubated in Phenol Red-free DMEM containing glutamine, antibiotics, and 10% dextran-coated charcoal-stripped FBS at 37 °C, 95% air and 5% CO_2_. At confluence, cells were treated with 1 x trypsin–EDTA and subcultured to use each experiment.

### Cell culture

To induce decidualization, hESCs were incubated with treatment medium (Phenol Red-free DMEM supplemented with glutamine, antibiotics and 2% dextran-coated charcoal-stripped FBS) containing dibutyryl-cAMP (0.5 mM) (Sigma, St Louis, MO, USA) for 96 h. The cells were then used for the experiments described below. cAMP is considered as a second messenger of progesterone for decidualization because progesterone increases intracellular cAMP concentrations in ESCs^31^. The concentration of cAMP (0.5 mM) and the period of incubation (96 h) used in this study were based on our previous report^31^. Cytochalasin D was added to the culture medium at the final concentration of 0.05 μM. The medium was changed every other day. We repeated the incubation with hESCs from more than three different individuals in each experimental procedure.

### Live cell imaging

hESCs were grown on gelatin-coated glass bottom dishes (MatTek, P35G-0-14-C, USA and MATSUNAMI, JAPAN) and placed in an incubation chamber stage (Tokai Hit) at 37 °C under 5% CO_2_ in air for live cell imaging. The fluorescence signals were observed using a LSM800 confocal microscope (Carl Zeiss, Germany), equipped with a laser module (405/488/561/640 nm) and GaAsP detector, using the same contrast, brightness, and exposure settings within the same experiments. Z-slice thickness was determined by using the optimal interval function in the ZEN software. Time series were performed with an interval of 10 min.

### Phalloidin staining

hESCs were grown on gelatin-coated glass bottom dishes. After treatment with cAMP, decidualized or control hESCs were fixed in 4% PFA/PBS at room temperature for 20 min, and were washed by PBS for 3 times. Cells were next treated with 0.5% triton X-100/PBS at room temperature for 20 min, followed by washing three times with 3% BSA/PBS. The samples were incubated in PBS with 100 nM Acti-stain™ 555 Phalloidin (PHDH1; Cytoskeleton, Inc., CO, USA) at room temperature for 25 min. Following three times washes by 3% BSA/PBS, samples were further stained with DAPI at room temperature for 10 min. The samples were washed with 3% BSA/PBS three times. The fluorescence signals were observed using a LSM800 microscope, equipped with a laser module (405/488/561/640 nm) and GaAsP detector, using the same contrast, brightness, and exposure settings. Z-slice thickness was determined by using the optimal interval function in the ZEN software.

### Immunofluorescence staining

hESCs were fixed in 4% PFA/PBS at room temperature for 20 min, and were washed by 0.01% BSA/PBS for 3 times. Samples were next treated with 0.5% triton X-100/PBS at room temperature for 20 min, followed by washing three times with 3% BSA/PBS. The samples were blocked in 3% BSA/PBS for 1 h at room temperature, then incubated with primary antibodies (Ki67; abcam, ab92742, diluted in 1:500 or Cofilin; Sigma, C8736, diluted in 1:1000) at 4 °C overnight. Following three times washes by 3% BSA/PBS, samples were further incubated in the dark with Alexa Fluor 568-labeled goat anti-rabbit IgG antibody (1:2,000; A11008, Thermo Fisher Scientific, Waltham, MA, USA) at room temperature for 1 hour. The samples were washed with 3% BSA/PBS three times and then mounted on slides using VECTASHIELD Mounting Medium containing DAPI. The fluorescence signals were observed using a LSM800 microscope, equipped with a laser module (405/488/561/640 nm) and GaAsP detector, using the same contrast, brightness, and exposure settings within the same experiments. Z-slice thickness was determined by using the optimal interval function in the ZEN software.

### Establishment of hESC lines stably expressing nAC-GFP, NLS-Actin^R62D^ or XPO6

The coding sequence of nAC-GFP was amplified by PCR with Prime STAR GXL DNA polymerase (TaKaRa, Ohtsu, Japan) using the pCS2-nAC-GFP vector as a template^26^. The amplicon was inserted at the multiple cloning site of pMXs-IRES-Blasticidin retroviral vector (Cell BIOLABS) by In-Fusion HD Cloning kit (TaKaRa). For retrovirus production, these vectors were co-transfected with packaging plasmids into HEK293 cells using Lipofectamine 2000 (Invitrogen). Retroviral supernatants were collected 48 h after transfection and passed through a 0.45 μm filter. The virus-containing supernatant was concentrated with PEG-it Virus Precipitation Solution (System Biosciences, Palo Alto, CA, USA). hESCs were seeded at 3 × 10^4^ cells in 12-well plates. The following day, virus concentrate was added to the medium with 8 μg/ml final concentration of Polybrene (Sigma). The plate was centrifuged at 800 × *g* for 60 min at 33 °C, washed with PBS and changed to a fresh medium. The hESC lines stably expressing nAC-GFP were established by selecting with 5 μg/ml blasticidin S (Thermo Fisher Scientific) for 4 days. Viruses to overexpress NLS-Actin^R62D^ and XPO6 were similarly generated and infected to hESCs as described above.

For the transient expression of nAC-GFP for Fig. 1a and Supplementary Fig. 1a, the pCS2-nAC-GFP vector^26^ was transfected to hESCs using Lipofectamine 3000 (Thermo fisher Scientific, L3000008) according to the vendor’s instruction.

### Real-time RT-PCR

Total RNA was isolated from the cultured cells with an NucleoSpin® RNA (TaKaRa). The RNA was reverse transcribed and real-time RT**-**PCR was performed with CFX384 Touch Real-Time PCR Detection System (Bio-Rad) as reported previously^67,68^ with sequence-specific primer sets (Supplementary Table 5). MRPL19 was used as an internal control.

### Western blotting

Western blotting was performed as reported previously^69,70^. Whole cell lysates were prepared using loading buffer reagents (Santa Cruz Biotechnology, Inc., Santa Cruz, CA, USA) without trypsin treatment. Nuclear and cytoplasmic lysates were prepared with NE-PER Nuclear and Cytoplasmic Extraction Reagents (Thermo fisher Scientific) according to the manufacturer’s protocol. Equal amounts of total protein were electrophoresed on a 10% SDS-polyacrylamide gel. The proteins were transferred to polyvinylidene difluoride membranes (ATTO, Tokyo, Japan). The membranes were blocked with blocking solution [5% skimmed milk with 0.1% Tween-20 dissolved in Tris-buffered saline (pH 7.5)], incubated with the first antibody for β-actin (abcam, ab6276, diluted in 1:10,000), cofilin (Sigma, C8736, diluted in 1:4000), XPO6 (BETHYL, A301-205A, diluted in 1:1000), C/EBPβ (Santa Cruz Biotechnology, sc-7962, diluted in 1:200), histone H2B (abcam, ab1790, diluted in 1:4000) and β-tubulin (Sigma, T4026, diluted in 1:1000), which were diluted in blocking solution, incubated with the peroxidase-conjugated secondary antibody diluted in blocking solution, visualized with the ECL-Western blotting detection system (Amersham, Aylesburg, UK) according to the manufacturer’s protocol. Western blot bands were quantified by ImageJ. Uncropped images of the immunoblots are provided in Supplementary Fig. 7.

### Lipid-mediated transfection of small interfering RNA (siRNA) duplexes

C/EBPβ ON-TARGET plus SMART pool and ON-TARGET plus Non-Targeting pool siRNA were purchased from Dharmacon (Lafayette, CO, USA). hESCs at 50% confluence were transfected with siRNA duplexes (20 nM) and RNAi MAX (Invitrogen) as we reported previously^49^. The medium was changed 5 h later. After 48 h of transfection, cells were incubated with or without cAMP for 96 h and then used for each experiment.

### RNA-sequencing (RNA-seq) Analysis

RNA extraction was performed by using NucleoSpin® RNA (TaKaRa) and transferred into 1×Reaction buffer from SMART-seq v4 Ultra Low Input RNA Kit (24888 N, Takara). SMART-seq library preparation was performed using SMART-seq v4 Ultra Low Input RNA Kit and Nextera DNA Sample Preparation Kit (FC-131-1024, illumine, San Diego, CA) according to the vendor’s instruction. Paired-end sequencing (50 bp + 25 bp) was done by using the NextSeq platform (Illumina). Raw reads were first filtered to get rid of low quality reads using Trimmomatic^71^. Reads of less than 20 bases and unpaired reads were also removed. Furthermore, removal of adaptor, polyA, polyT and polyG sequences were performed using Trim Galore! (https://www.bioinformatics.babraham.ac.uk/projects/trim_galore/). For mapping of reads, trimmed reads were first aligned to the human genome hg19 using STAR aligner^72^. Gene expression values were calculated as “fragments per kilobase of exon unit per million mapped reads” (FPKM). Gene counts in triplicate were used to identify differentially expressed genes (DEGs, fulfilling the following criteria: *p* adj < 0.05) using DESeq2^73^. Each gene list was further subjected to gene ontology (GO) analysis, KEGG pathway analysis (https://david.ncifcrf.gov/) and Ingenuity Pathway Analysis (IPA; QIAGEN, Redwood City, CA). Using IPA, enriched canonical pathways, upstream transcriptional regulators, and diseases and biological functions were investigated.

### Statistics and reproducibility

All of the statistical methods are described in the figure legends. Fisher’s exact test was used for Supplementary Fig. 2 to evaluate the differences of the proportion of cells with nuclear F-actin network between control and cAMP-treated samples. Two-sided student’s *t* test was used for Figs. 3f, 7a, 7b and Supplementary Fig. 1b, 1c, 1d. For other analyses to evaluate the differences between groups, one-way ANOVA followed by a Tukey-Kramer test was used. Differences were considered significant at *P* < 0.05.

### Reporting summary

Further information on research design is available in the Nature Portfolio Reporting Summary linked to this article.

### Supplementary information

Supplementary information

Description of Additional Supplementary Files

Supplementary data

Supplementary Movie 1.

Supplementary Movie 2.

Supplementary Movie 3.

Reporting Summary

## Data Availability

RNA-seq data were deposited into the Gene Expression Omnibus database under accession number GSE200945. All data supporting the findings of this study are available within the paper and its Supplementary Information. The source data behind the graphs in the paper can be found in Supplementary Data. All the other data are available from the corresponding author on reasonable request.

## Code availability

RNA-seq data were deposited into the Gene Expression Omnibus database under accession number GSE200945. All data supporting the findings of this study are available within the paper and its Supplementary Information. The source data behind the graphs in the paper can be found in Supplementary Data. All the other data are available from the corresponding author on reasonable request.
