# Supplementary material for: Nuclear actin assembly is an integral part of decidualization in human endometrial stromal cells
Source: Commun Biol. 2024 Jul 11;7:830. doi: 10.1038/s42003-024-06492-z (PMC11239864; doi:10.1038/s42003-024-06492-z)
Supplement: Supplementary file 1 — Supplementary information [file 42003_2024_6492_MOESM1_ESM.pdf]

Supplementary information

Nuclear actin assembly is an integral part of decidualization in human endometrial stromal cells

Isao Tamura, Kei Miyamoto, Taishi Fujimura, Yuichiro Shirafuta, Yumiko Mihara, Ryo Maekawa, Toshiaki Taketani, Shun Sato, Kazuya Matsumoto, Hiroshi Tamura, Norihiro Sugino

Supplementary Figures 1-7
Supplementary Movies 1-3
Supplementary Tables 1-5

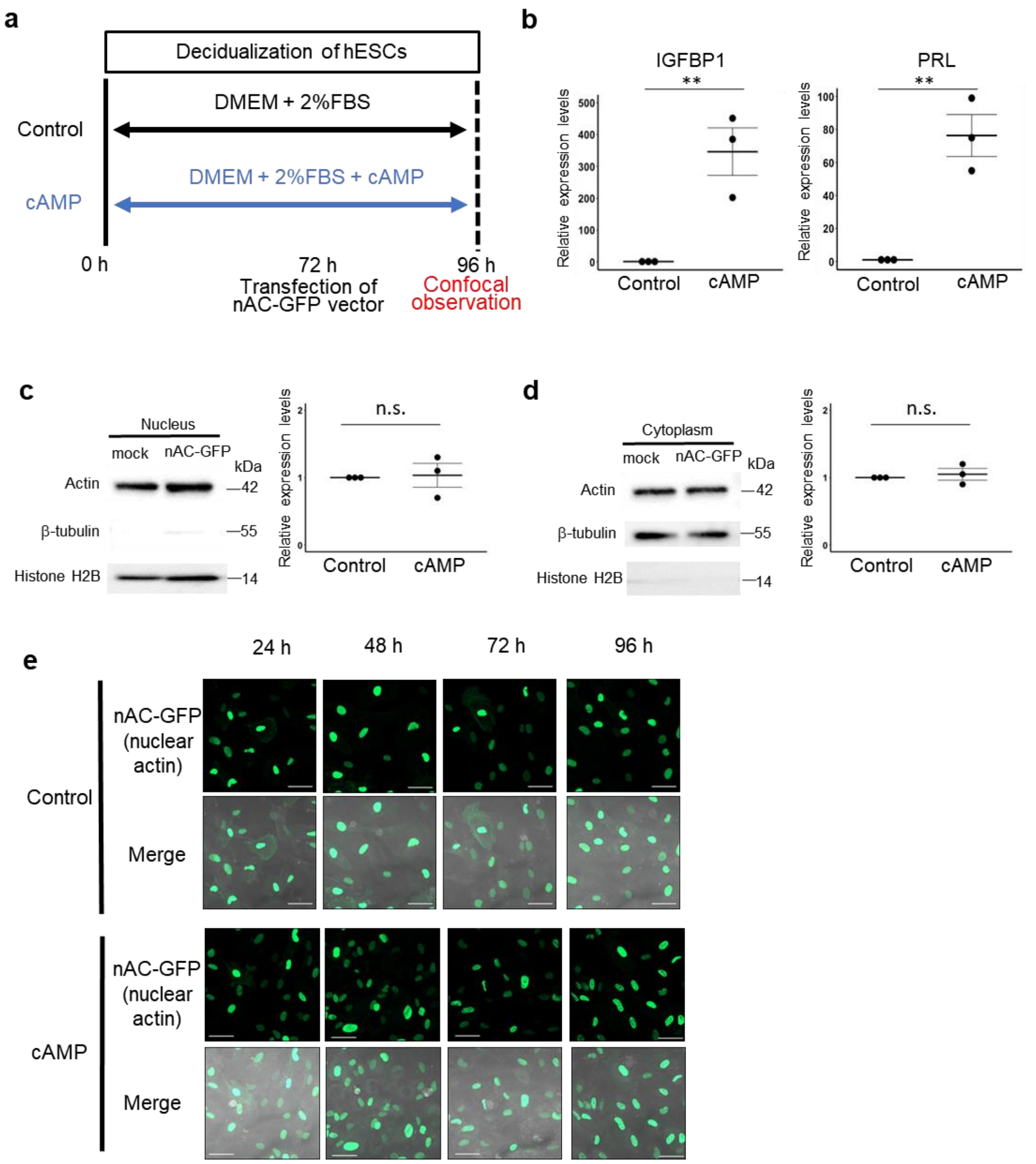

Supplementary Fig. 1: The networked structure of nuclear actin is formed in hESCs during decidualization.

a, A schematic diagram to capture nuclear actin assembly during decidualization of hESC cells that were transiently expressed with nAC-GFP, a nuclear actin probe. **b,** RT-qPCR analyses of decidualization markers (IGFBP1 and PRL) of hESCs that were transiently expressed with nAC-GFP. Cells were treated with or without cAMP for 96 h. Relative expression levels to control untreated hESCs are shown. Mean ± SE of three independent experiments. Each data point is indicated as a dot. **, $P<0.01$ (Student's t-test). **c,** Overexpression of nAC-GFP did not affect the nuclear actin levels in non-decidualized ESCs. hESCs were overexpressed with mCherry (as a mock control) or nAC-GFP. Nuclear actin levels were examined. Representative images of

western blot analyses against β-actin, β-tubulin and histone H2B are shown. Mean ± SE of three independent experiments. Each data point is indicated as a dot. n.s. represents not significant (Student's t-test). **d,** Overexpression of nAC-GFP did not affect the cytoplasmic actin levels in decidualized ESCs. hESCs were overexpressed with mCherry (as a mock control) or nAC-GFP, and were treated with or without cAMP for 96 h. Cytoplasmic actin levels were examined. Representative images of western blot analyses against β-actin, β-tubulin and histone H2B are shown. Mean ± SE of three independent experiments. Each data point is indicated as a dot. n.s. represents not significant (Student's t-test). **e,** Representative confocal images of hESC cells stably expressing nAC-GFP during decidualization. Times after cAMP supplementation are indicated above. As a control, hESCs without cAMP addition were examined. Merged images of GFP channels and DIC are shown in Merge. Scale bars, 50 μm. Three independent experiments were repeated.

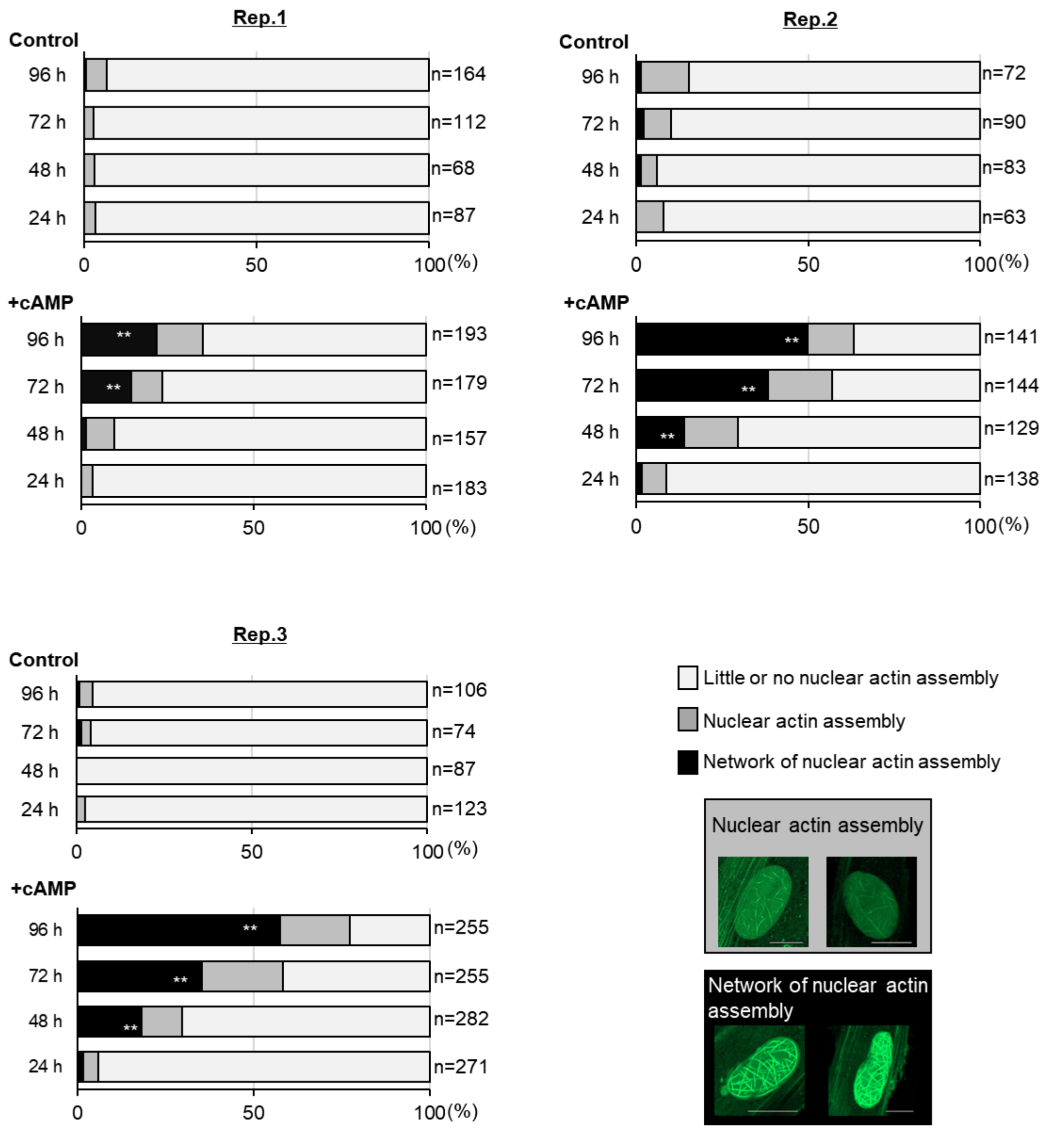

Supplementary Fig. 2: Proportions of cells that showed nuclear actin assembly or interconnected network of nuclear actin filaments.

Times after cAMP supplementation are indicated at each bar. The numbers of cells examined are indicated next to each bar. Ratios of cells that showed the network of nuclear actin assembly to total cells were compared between control cells and cAMP-treated cells at each time point. Representative images for cells showing nuclear actin assembly and those showing the network of nuclear actin assembly are indicated at the bottom, right. Statistical significance was calculated using the Fisher's exact test. ** $p < 0.01$ vs. control (Fisher's exact test).

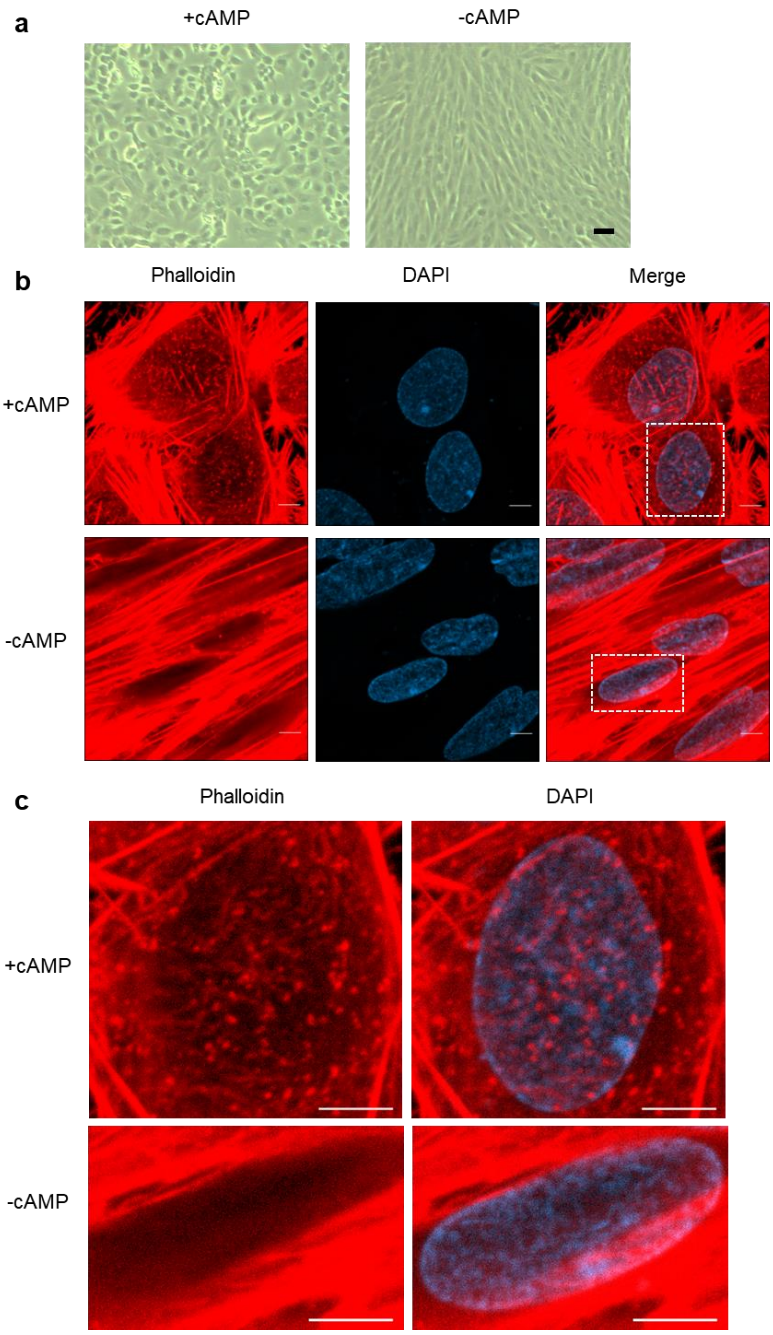

Supplementary Fig. 3: Endogenous nuclear F-actin in decidualized hESCs.

a, A bright filed image of hESCs after 4 days of cAMP treatment (+cAMP). As a control, hESCs were cultured in DMEM with 2% FBS without cAMP supplementation (-cAMP). Scale bar, 50 µm. **b, c,** Endogenous F-actin was stained with phalloidin in decidualized (+cAMP) and non-decidualized hESCs (-cAMP). DNA was visualized by DAPI. Enlarged images in (**c**) correspond to the areas shown by white-dotted squares in (**b**). Scale bars, 5 µm.

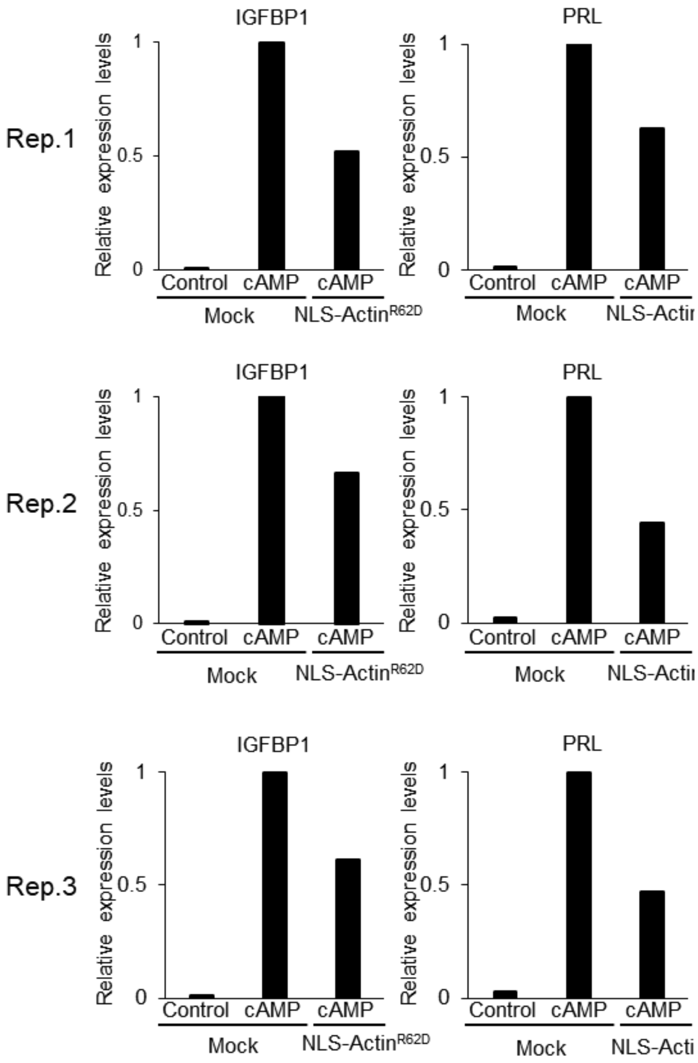

Supplementary Fig. 4: Expression of decidualization markers is downregulated by forced disassembly of nuclear actin.

RT-qPCR analyses of decidualization markers (IGFBP1 and PRL) of three hESC lines that were subjected to RNA-seq analysis in Fig. 4. hESCs were overexpressed with mCherry (as a mock control) or NLS-Actin^{R62D}, and were treated with or without cAMP for 96 h. hESCs derived from different women are used. All cell lines similarly achieved the upregulation of decidualization marker genes. Relative expression levels to cAMP-treated mock hESCs are shown.

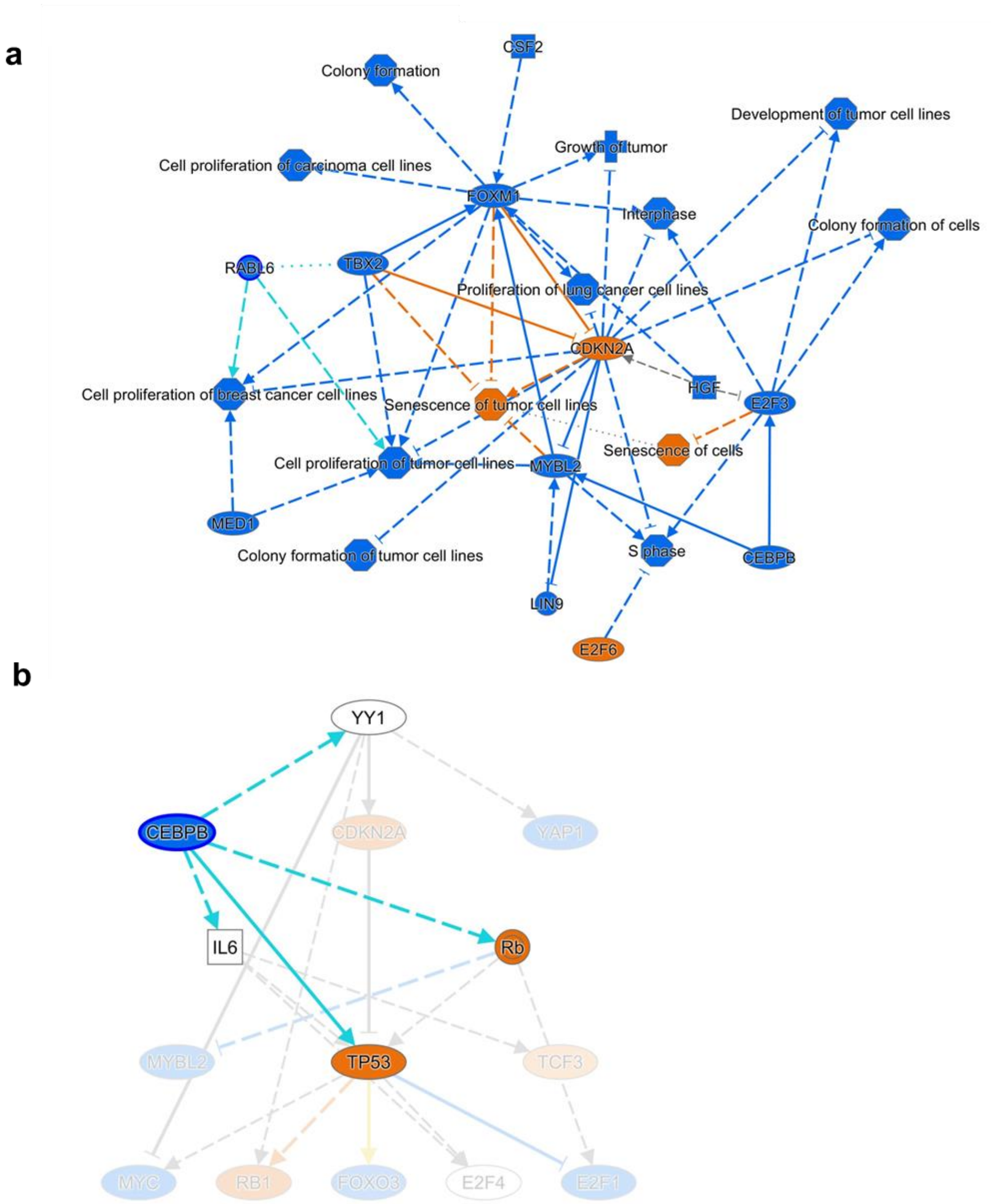

Supplementary Fig. 5: Molecular networks associated with nuclear actin assembly-regulated decidualization genes.

a, IPA predicted genes and molecular networks that are closely related to nuclear actin assembly-regulated decidualization genes (Fig. 4b). Blue colors indicate predicted inhibition, while orange colors show predicted activation. **b,** Relationship between C/EBPβ and predicted upstream regulators for nuclear actin assembly-regulated decidualization genes is depicted by IPA.

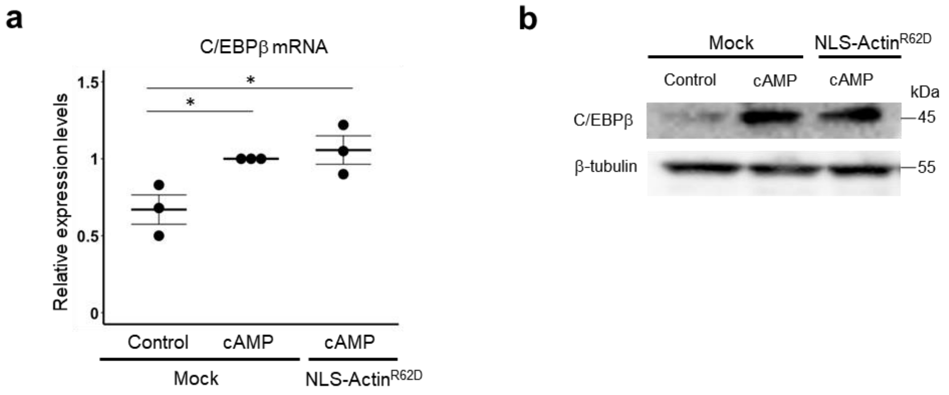

Supplementary Fig. 6: Effect of overexpressing NLS-Actin^{R62D} on C/EBP β expression.

a, The effect of overexpressing NLS-Actin^{R62D} on C/EBPβ mRNA expression. The expression levels of C/EBPβ were extracted from RNA-seq data (Fig. 4). Relative expression levels to cAMP-treated mock hESCs are shown. Explanations to each sample are described in Fig. 4a. Mean ± SE of three independent experiments. Each data point is indicated as a dot. *, $P<0.05$ (Tukey-Kramer test). **b,** Representative images of western blot analyses of cAMP-treated hESCs with or without overexpression of NLS-Actin^{R62D}. hESC were overexpressed with mCherry (as a mock control) or NLS-Actin^{R62D}, and were treated with or without cAMP for 96 h. Antibodies against C/EBPβ and β-tubulin are used.

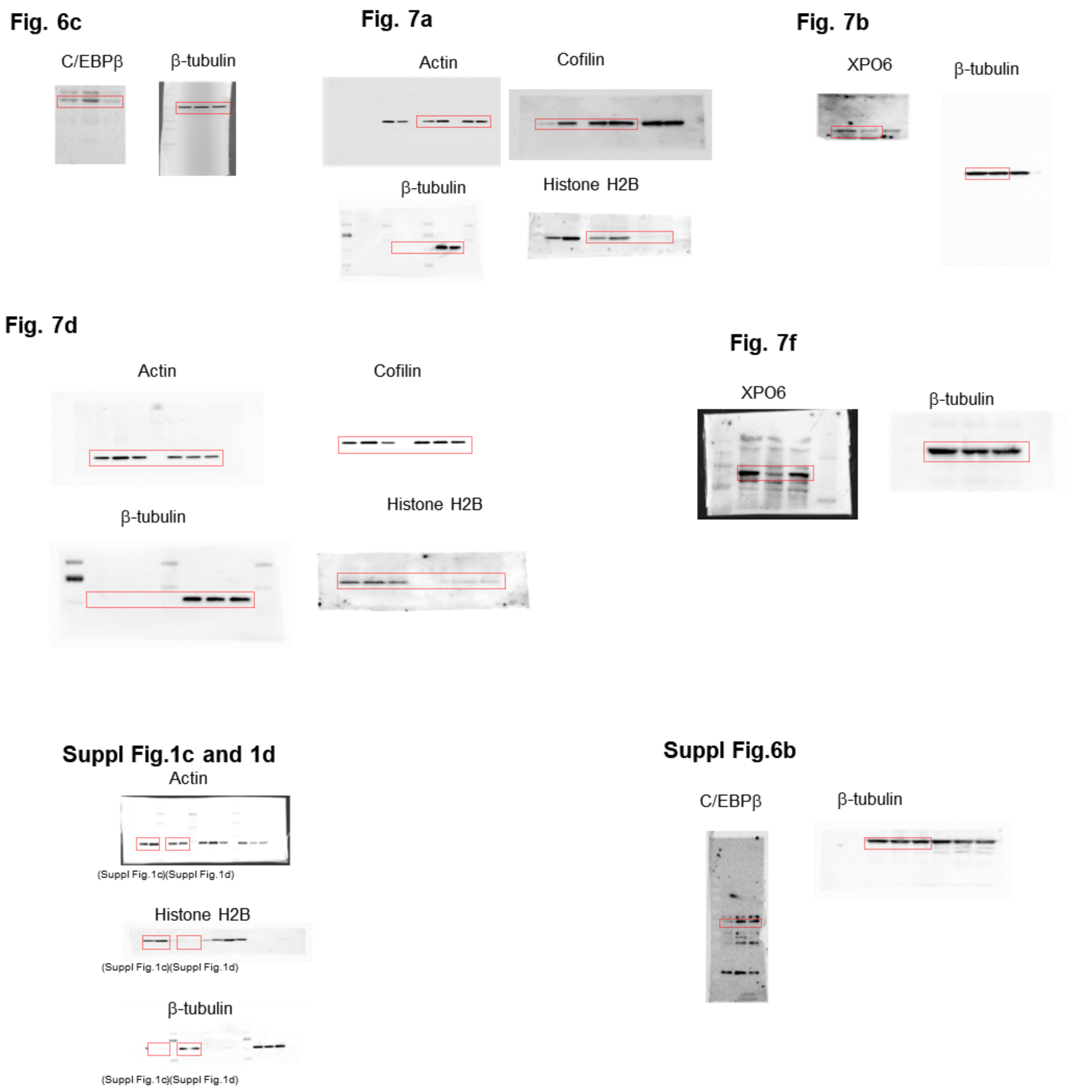

Supplementary Fig. 7: Uncropped images of the immunoblots of all relevant figures.

Supplementary Movie Legends

Supplementary Movie 1. Nuclear F-actin formation during decidualization

Images were taken from 57 hours after cAMP supplementation, and each image was taken every 10 min for 730 min.

Supplementary Movie 2. A process of forming thick nuclear actin filaments during decidualization

Images were taken from 57 hours after cAMP supplementation, and each image was taken every 10 min for 730 min.

Supplementary Movie 3. Disappearance of nuclear F-actin

Nuclear F-actin in decidualized cells was disassembled 3 days after cAMP withdraw. Images were taken from 72 hours after cAMP withdrawal, and each image was taken every 10 min for 310 min.

Supplementary Table 1. List of nuclear actin assembly-regulated decidualization genes

ABHD2	CAP2	EGR2	KRT34	PHLDA2	SBF2
ABI3BP	CAPN12	EMP1	KRT8	PKD2	SERTAD4-AS1
ABRACL	CCNA2	ENAH	KRTAP1-5	PKMYT1	SH2D5
AC006483.1	CCNB1	EPG5	KRTAP2-3	PKP2	SH3BGRL
AC087645.1	CCNB2	ESYT1	LANCL3	PLEKHO1	SHCBP1
AC107016.1	CCND2	EZR	LASP1	PLK1	SKA3
AC107016.2	CCNE2	F2RL1	LFNG	PLK4	SLC16A2
AC124789.1	CD44	FADS3	LIG1	POC1A	SLC28A3
ACOT7	CD82	FAM111B	LINC00152	POU2F2	SLC35F5
ACTBP2	CDC42BPA	FAM19A2	LINC00704	PPFIBP1	SLC43A2
ACTR3	CDCA8	FAM83D	LRRC2	PPME1	SOD3
AFAP1L1	CDCP1	FGD4	LRRFIP1	PRDX6	SOGA2
AFAP1L2	CDK1	FHDC1	MACROD2	PRELID2	SORT1
AJUBA	CDK15	FIBCD1	MAN1C1	PRKAG2	SPC25
AKAP2	CENPE	FLT1	MAP2K3	PRKCDBP	SRD5A1
ANKRD13A	CENPF	FMN2	MCAM	PRPS1	SRFBP1
ANO1	CENPK	FN3KRP	MCM2	PRR11	STMN1
ANXA1	CEP55	FOXM1	MCM4	PRR5L	SYDE2
ANXA2	CEP78	FRMD4A	MCM7	PRSS23	SYPL2
ANXA2P2	CHDH	FZD2	MDGA1	PTCHD4	TBC1D1
APPL2	CHRM2	GAB1	MELK	PTPLA	TCF19
ARHGAP11A	CLDN1	GALNT3	MFGE8	PTPRR	TDO2
ARHGAP18	CLIC1	GFRA1	MGLL	PXN	TLN1
ARNT2	CLSPN	GINS1	MKI67	RAB3B	TMEM173
ARRDC4	COBLL1	GINS2	MKX	RAD51	TMEM40
ASAP2	COL11A1	GLIPR2	MLF1IP	RAD51AP1	TNIK
ASF1B	CORIN	GLS	MTFR2	RASGRP3	TPST2
ASPN	CPED1	GNB4	MYBL2	RBM3	TPX2
ATAD5	CRIP2	GPR176	MYL6	RGS5	TRIB2
ATP10D	CTC-512J14.7	H2AFJ	MYO1D	RHOBTB1	TRIM47
ATP11C	CTD-2334D19.1	HEBP1	MYOZ1	RIMS1	TRIM55
ATP2B1	CYP2U1	HECW2	NAV2	RNASEH2A	TRIM59
AURKB	CYR61	HJURP	NCAPH	RP11-109L13.1	TRIP13
B3GALT2	DAPK3	HMGA1	NEO1	RP11-121L10.3	TRPV2
B4GALT6	DCLK2	HTR2B	NFASC	RP11-161H23.5	TUBA1B
BAIAP2L1	DEPDC1	ICA1L	NIPAL3	RP11-173B14.5	TUBA1C
BARD1	DKK3	IGJ	NMT2	RP11-344E13.3	TXNIP
BBC3	DLGAP5	IQGAP3	NT5DC3	RP11-386G11.10	TYMS
BEX1	DOPEY2	ITGA11	NUSAP1	RP11-474B16.1	UBA7
BID	DPF3	JAG1	ORAOV1	RP11-517I3.1	UBE2C
BIRC5	DSTN	JAZF1	ORC6	RP11-527D7.1	UBXN11
BMPR1B	DTNA	KCNIP4	PALM2	RP11-603J24.18	UCP2
BUB1	DUSP5	KHDRBS3	PALM2-AKAP2	RP11-818F20.5	VAT1
C12orf60	DUSP6	KIAA0101	PAQR4	RP11-84A19.3	WDFY1
C1QTNF9B	DYNLT3	KIAA1462	PBK	RP3-388N13.2	WEE1
C1QTNF9B-AS1	E2F7	KIF13A	PCK2	RP3-412A9.10	ZEB2
C2orf27A	EBF1	KIF20A	PDCL3	RRAD	ZFHX4
C7orf69	EDIL3	KIF22	PDE4DIP	RRAS	ZNF106
CAMK1D	EDN1	KIF23	PDLIM1	RTN4	ZNF367
CAMK4	EFNA5	KLF5	PEA15	S100A11	
CAP1	EFNB2	KRT18	PFKP	SAMD4A	

Supplementary Table 2. List of enriched gene ontology and pathway terms in nuclear actin assembly-regulated decidualization genes

term_ID	GO term	p value	Gene symbol
GO:0051301	cell division	7.21E-08	LIG1, UBE2C, DYNLT3, CDCA8, SKA3, NCAPH, CCNA2, CENPE, CCNB2, TUBA1C, TPX2, CENPF, TUBA1B, WEE1, CCNB1, CCND2, CCNE2, CDK1, BIRC5, FAM83D, BUB1, SPC25
GO:0007067	mitotic nuclear division	2.05E-07	DYNLT3, PLK1, KIF22, PKMYT1, SKA3, AURKB, CCNA2, CCNB2, TPX2, CENPF, WEE1, PBK, CDK1, BIRC5, FAM83D, BUB1, CEP55, SPC25
GO:0051726	regulation of cell cycle	3.47E-07	PLK1, PKMYT1, FOXM1, CCNB2, CENPF, WEE1, CCNB1, KIAA0101, CCNE2, MYBL2, CLIC1, PRR11, CDK15
GO:0000086	G2/M transition of mitotic cell cycle	1.02E-06	PLK4, PLK1, PKMYT1, FOXM1, CCNB2, TPX2, WEE1, CCNB1, MELK, CDK1, BIRC5, AJUBA, CEP78
GO:0006268	DNA unwinding involved in DNA replication	9.54E-06	RAD51, MCM7, HMGA1, MCM4, MCM2
GO:0007062	sister chromatid cohesion	2.39E-05	CENPE, CENPF, PLK1, CENPK, CDCA8, BIRC5, KIF22, BUB1, AURKB, SPC25
GO:0098609	cell-cell adhesion	6.02E-05	ANXA2, ARHGAP18, PRDX6, RTN4, PDLIM1, CCNB2, PPFIBP1, LASP1, BAIAP2L1, KRT18, PPME1, COBLL1, LRRFIP1, S100A11, PFKP
GO:0000281	mitotic cytokinesis	6.35E-05	PLK1, STMN1, NUSAP1, KIF23, KIF20A, CEP55
GO:0006260	DNA replication	1.15E-04	BARD1, GINS2, RNASEH2A, ORC6, MCM7, KIAA0101, LIG1, CDK1, MCM4, CLSPN, MCM2
GO:0008283	cell proliferation	1.31E-04	TCF19, MCM7, PLK1, GAB1, EMP1, MKI67, TYMS, CYR61, AURKB, TPX2, CENPF, MELK, CDK1, FAM83D, BUB1, DLGAP5, APPL2
GO:0000082	G1/S transition of mitotic cell cycle	1.46E-04	ORC6, MCM7, CCNE2, CDK1, MCM4, PKMYT1, TYMS, IQGAP3, MCM2
GO:0051310	metaphase plate congression	6.57E-04	CENPE, CENPF, KIF22, FAM83D
GO:0000910	cytokinesis	7.27E-04	PLK1, KIF13A, DAPK3, BIRC5, KIF23, KIF20A
GO:0006270	DNA replication initiation	0.0013	ORC6, MCM7, CCNE2, MCM4, MCM2
GO:0007080	mitotic metaphase plate congression	0.0022	CENPE, CCNB1, CDCA8, KIF22, CEP55
GO:0043087	regulation of GTPase activity	0.0029	SYDE2, FGD4, EFNA5, AJUBA, SBF2, IQGAP3
GO:0007346	regulation of mitotic cell cycle	0.0029	AFAP1L2, DYNLT3, PLK1, DAPK3, BIRC5
GO:0006468	protein phosphorylation	0.0037	PLK4, CAMK1D, PLK1, DAPK3, PRKAG2, PKMYT1, CDC42BPA, AURKB, CAMK4, PBK, BIRC5, TNIK, BMPR1B, TRIB2, BUB1, CDK15
GO:0051439	regulation of ubiquitin-protein ligase activity involved in mitotic cell cycle	0.0047	CCNB1, UBE2C, PLK1, CDK1
GO:0008360	regulation of cell shape	0.0051	FGD4, ANXA1, PALM2, DAPK3, ARHGAP18, PLEKHO1, EZR, PALM2-AKAP2
GO:0051983	regulation of chromosome segregation	0.0059	MKI67, BUB1, AURKB
GO:0030071	regulation of mitotic metaphase/anaphase	0.0075	CENPE, UBE2C, PLK1
GO:0009653	anatomical structure morphogenesis	0.0123	EFNB2, KRT18, LIG1, MCAM, CYR61, DKK3
GO:0008154	actin polymerization or depolymerization	0.0133	CAP1, DSTN, CAP2
GO:0097284	hepatocyte apoptotic process	0.0156	KRT18, KRT8, BID
GO:0042493	response to drug	0.0163	CENPF, CCNB1, RAD51, ANXA1, MCM7, SRD5A1, CDK1, HTR2B, TXNIP, TYMS, DUSP6
GO:0007059	chromosome segregation	0.0188	CENPE, CENPF, HJURP, SKA3, SPC25
GO:0043066	negative regulation of apoptotic process	0.0198	BARD1, ARNT2, ANXA1, CAMK1D, PLK1, HTR2B, FMN2, CYR61, KRT18, CCND2, UCP2, CDK1, BIRC5, CD44
GO:0000226	microtubule cytoskeleton organization	0.0217	TUBA1B, WEE1, CDK1, DCLK2, BIRC5
GO:0000187	activation of MAPK activity	0.0222	MAP2K3, DUSP5, PEA15, CDK1, IQGAP3, DUSP6
GO:0030334	regulation of cell migration	0.0248	JAG1, GAB1, PHLDA2, AJUBA, RTN4
GO:0030033	microvillus assembly	0.0261	KLF5, TNIK, EZR
GO:0007077	mitotic nuclear envelope disassembly	0.0279	CCNB2, CCNB1, PLK1, CDK1
GO:0030705	cytoskeleton-dependent intracellular transport	0.0291	TUBA1C, TUBA1B, KIF13A
GO:0031145	anaphase-promoting complex-dependent	0.0306	CCNB1, UBE2C, PLK1, CDK1, AURKB
GO:0045860	positive regulation of protein kinase activity	0.0330	MAP2K3, CENPE, PRKAG2, CYR61
GO:0031532	actin cytoskeleton reorganization	0.0330	ANXA1, TNIK, EZR, CDC42BPA
GO:0031100	organ regeneration	0.0330	CCNA2, PRPS1, CDK1, MKI67
GO:0007018	microtubule-based movement	0.0331	CENPE, KIF13A, KIF23, KIF20A, KIF22
GO:0007094	mitotic spindle assembly checkpoint	0.0354	CENPF, PLK1, BUB1
GO:0006974	cellular response to DNA damage stimulus	0.0371	BARD1, RAD51, MCM7, KIAA0101, ATAD5, FMN2, MACROD2, BBC3
GO:0001934	positive regulation of protein phosphorylation	0.0420	CCND2, ANXA2, PRR5L, TNIK, EFNA5, CYR61
GO:0007049	cell cycle	0.0446	KRT18, CCND2, HJURP, TXNIP, FOXM1, AURKB, APPL2, MCM2
GO:0007088	regulation of mitotic nuclear division	0.0495	DAPK3, MKI67, PKMYT1

term_ID	Pathway term	p value	Gene symbol
hsa04110	Cell cycle	9.87E-09	MCM7, PLK1, PKMYT1, CCNA2, CCNB2, WEE1, CCNB1, ORC6, CCND2, CCNE2, CDK1, MCM4, BUB1, MCM2
hsa04115	p53 signaling pathway	3.17E-05	CCNB2, CCNB1, CCND2, CD82, CCNE2, CDK1, BID, BBC3
hsa04914	Progesterone-mediated oocyte maturation	0.0012	CCNA2, CCNB2, CCNB1, PLK1, CDK1, PKMYT1, BUB1
hsa03030	DNA replication	0.0014	RNASEH2A, MCM7, LIG1, MCM4, MCM2
hsa04114	Oocyte meiosis	0.0040	CCNB2, CCNB1, CCNE2, PLK1, CDK1, PKMYT1, BUB1
hsa04068	FoxO signaling pathway	0.0098	PLK4, CCNB2, CCNB1, CCND2, PLK1, PRKAG2, PCK2

Supplementary Table 3. List of enriched gene ontology terms in the 63 genes

term_ID	GO term	p value	Gene symbol
GO:0030198	extracellular matrix organization	0.0020	VCAN, LAMA2, COL5A1, COL14A1, FBN1
GO:0035148	tube formation	0.0217	BCL2L11, ATOH8

Supplementary Table 4. List of candidate upstream factors identified by IPA analysis

Upstream regulator	Absolute value of z-score	p value
CEBPB	4.491	3.E-13
MITF	3.302	2.E-07
MYC	3.275	2.E-04
FOXM1	3.227	3.E-10
TBX2	3.162	8.E-11
NUPR1	3.13	2.E-10
CDKN2A	3.06	2.E-09
E2F3	2.804	3.E-07
TCF7L2	2.753	0.00556
YAP1	2.588	4.E-06
FOXO1	2.563	8.E-05
TAL1	2.449	5.E-04
MED1	2.449	0.00391
E2F1	2.368	5.E-08
E2F6	2.236	2.E-07
MYBL2	2.205	8.E-07
CCND1	2.158	4.E-18
RB1	2.105	3.E-11
POU2F2	2	6.E-04
TCF4	2	0.00396
GATA4	1.994	0.00765
KDM5B	1.987	0.00213
SRF	1.982	0.00231
ARNT	1.96	2.E-04
RBL1	1.939	2.E-04
TP53	1.912	1.E-07
SMARCB1	1.633	1.E-06
TRPS1	1.633	4.E-04
FOXO3	1.537	2.E-10
WBP2	1.455	0.00206
RUNX2	1.408	2.E-04
GLI1	1.112	2.E-04
TCF3	1	1.E-05
HDAC2	1	5.E-04
ZNF217	1	0.00188
TP73	0.574	0.00167
SOX2	0.198	0.0064
KLF5	0.089	3.E-04
KLF4	0.045	0.0062
IKZF1	0	7.E-06
CREB1	0	0.00673

Supplementary Table 5. Primer sequences used in this study

	Forward	Reverse
IGFBP-1	CGAAGGCTCTCCATGTCACCA	TGTCTCCTGTGCCTTGGCTAAAC
PRL	AAAGGATCGCCATGGAAAG	GCACAGGAGCAGGTTTGAC
MRPL19	GAATGTTATCGAAGGACAAGGT	CAGGAAGGGCATCTCGTAAG
