# Supplementary material for: Nuclear actin assembly is an integral part of decidualization in human endometrial stromal cells
Source: Commun Biol. 2024 Jul 11;7:830. doi: 10.1038/s42003-024-06492-z (PMC11239864; doi:10.1038/s42003-024-06492-z)
Supplement: Supplementary file 2 — Description of Additional Supplementary Files [file 42003_2024_6492_MOESM2_ESM.pdf]

Description of Additional Supplementary Files

File name: Supplementary Movie 1.

Description; Nuclear F-actin formation during decidualization.

File name: Supplementary Movie 2.

Description; A process of forming thick nuclear actin filaments during decidualization.

File name: Supplementary Movie 3.

Description; Disappearance of nuclear F-actin

File name: Supplementary Data.

Description. The source data behind the graphs in the paper.
