# Supplementary material for: Nuclear actin assembly is an integral part of decidualization in human endometrial stromal cells
Source: Commun Biol. 2024 Jul 11;7:830. doi: 10.1038/s42003-024-06492-z (PMC11239864; doi:10.1038/s42003-024-06492-z)
Supplement: Supplementary file 7 — Reporting Summary [file 42003_2024_6492_MOESM7_ESM.pdf]

nature portfolio

Corresponding author(s): Isao Tamura and Kei Miyamoto

Last updated by author(s): Jun 5, 2024

Reporting Summary

Nature Portfolio wishes to improve the reproducibility of the work that we publish. This form provides structure for consistency and transparency in reporting. For further information on Nature Portfolio policies, see our Editorial Policies and the Editorial Policy Checklist.

Statistics

For all statistical analyses, confirm that the following items are present in the figure legend, table legend, main text, or Methods section.

n/a	Confirmed	
☐	☒	The exact sample size (*n*) for each experimental group/condition, given as a discrete number and unit of measurement
☐	☒	A statement on whether measurements were taken from distinct samples or whether the same sample was measured repeatedly
☐	☒	The statistical test(s) used AND whether they are one- or two-sided<br>*Only common tests should be described solely by name; describe more complex techniques in the Methods section.*
☐	☒	A description of all covariates tested
☐	☒	A description of any assumptions or corrections, such as tests of normality and adjustment for multiple comparisons
☐	☒	A full description of the statistical parameters including central tendency (e.g. means) or other basic estimates (e.g. regression coefficient) AND variation (e.g. standard deviation) or associated estimates of uncertainty (e.g. confidence intervals)
☐	☒	For null hypothesis testing, the test statistic (e.g. F, t, r) with confidence intervals, effect sizes, degrees of freedom and P value noted<br>*Give P values as exact values whenever suitable.*
☒	☐	For Bayesian analysis, information on the choice of priors and Markov chain Monte Carlo settings
☒	☐	For hierarchical and complex designs, identification of the appropriate level for tests and full reporting of outcomes
☒	☐	Estimates of effect sizes (e.g. Cohen's *d*, Pearson's *r*), indicating how they were calculated

Our web collection on statistics for biologists contains articles on many of the points above.

Software and code

Policy information about availability of computer code

Data collection	The fluorescence signals were observed using a LSM800 confocal microscope (Carl Zeiss, Germany). RT-PCR was performed with CFX384 Touch Real-Time PCR Detection System (Bio-Rad). RNA-sequence data was collected with NextSeq platform (Illumina).
Data analysis	Regarding RNA-seq analysis, gene counts in triplicate were used to identify differentially expressed genes (DEGs, fulfilling the following criteria: padj<0.05) using DESeq2. Each gene list was further subjected to gene ontology (GO) analysis, KEGG pathway analysis (https://david.ncifcrf.gov/) and Ingenuity Pathway Analysis (IPA; QIAGEN, Redwood City, CA). Using IPA, enriched canonical pathways, upstream transcriptional regulators, and diseases and biological functions were investigated.

For manuscripts utilizing custom algorithms or software that are central to the research but not yet described in published literature, software must be made available to editors and reviewers. We strongly encourage code deposition in a community repository (e.g. GitHub). See the Nature Portfolio guidelines for submitting code & software for further information.

Data

Policy information about availability of data

All manuscripts must include a data availability statement. This statement should provide the following information, where applicable:

- Accession codes, unique identifiers, or web links for publicly available datasets
- A description of any restrictions on data availability
- For clinical datasets or third party data, please ensure that the statement adheres to our policy

Bulk RNA-sequencing data have been deposited at the Gene Expression Omnibus (GEO) public repository under accession GEO: GSE200945. [https://www.ncbi.nlm.nih.gov/geo/query/acc.cgi?acc=GSE200945].

Field-specific reporting

Please select the one below that is the best fit for your research. If you are not sure, read the appropriate sections before making your selection.

☒ Life sciences ☐ Behavioural & social sciences ☐ Ecological, evolutionary & environmental sciences

For a reference copy of the document with all sections, see nature.com/documents/nr-reporting-summary-flat.pdf

Life sciences study design

All studies must disclose on these points even when the disclosure is negative.

Sample size	We repeated the incubation with human cells from more than three different individuals in each experimental procedure.
Data exclusions	No data were excluded.
Replication	Each experiment was done at least with n>3. We confirmed that all attempts at replicon are successful.
Randomization	No formal randomization techniques were applied. Cultured cells were allocated randomly to experiments and processed in an arbitrary order.
Blinding	The investigators were blinded to group allocation during data collection.

Reporting for specific materials, systems and methods

We require information from authors about some types of materials, experimental systems and methods used in many studies. Here, indicate whether each material, system or method listed is relevant to your study. If you are not sure if a list item applies to your research, read the appropriate section before selecting a response.

Materials & experimental systems

n/a	Involved in the study	
☐	☒	Antibodies
☒	☐	Eukaryotic cell lines
☒	☐	Palaeontology and archaeology
☒	☐	Animals and other organisms
☐	☒	Human research participants
☒	☐	Clinical data
☒	☐	Dual use research of concern

Methods

n/a	Involved in the study	
☒	☐	ChIP-seq
☒	☐	Flow cytometry
☒	☐	MRI-based neuroimaging

Antibodies

Antibodies used	For immunofluorescence staining, antibodies against Ki67 (1:500 dilution, Cat No. ab92742, abcam), Cofilin (1:1000 dilution, Cat No. C8736, Sigma) and Alexa Fluor 568-labeled goat anti-rabbit IgG antibody (1:2000 dilution, Cat No. A11008, Thermo Fisher Scientific) were used. For western blotting analysis, antibodies against ⍰-actin (1:1000 dilution, Cat No. ab6276, abcam), XPO6 (1:1000 dilution, Cat No. A301-205A, BETHYL), C/EBP⍰ (1:200 dilution, Cat No. sc-7962, Santa Cruz Biotechnology), histone H2B (1:1000 dilution, Cat No. ab1790, abcam) and ⍰-tubulin (1:1000 dilution, Cat No. T4026, Sigma) were used.
Validation	Validation information is found in each manufacture's websites.<br>abcam: https://www.abcam.co.jp/ki67-antibody-epr3610-ab92742.html<br>https://www.abcam.co.jp/beta-actin-antibody-ac-15-ab6276.html<br>https://www.abcam.co.jp/histone-h2b-antibody-chip-grade-ab1790.html<br>Sigma: https://www.sigmaaldrich.com/JP/ja/product/sigma/c8736<br>https://www.sigmaaldrich.com/JP/ja/product/sigma/t4026<br>Thermo Fisher Scientific: https://www.thermofisher.com/antibody/product/Goat-anti-Rabbit-IgG-H-L-Cross-Adsorbed-Secondary-Antibody-Polyclonal/A-11008<br>BETHYL: https://www.fortislife.com/cms/files/A301-205A-1.pdf<br>Santa Cruz Biotechnology: https://datasheets.scbt.com/sc-7962.pdf

Human research participants

Policy information about studies involving human research participants

Population characteristics	Human endometrial tissues were obtained at hysterectomy from patients with a normal menstrual cycle, aged 40-45 years,

Population characteristics	who underwent surgery for myoma uteri or early stage cervical cancer. The patients were not on hormonal therapy at the time of surgery.
Recruitment	The methods were carried out in accordance with the approved guidelines. All patients gave their informed consent within written treatment contract on admission and therefore prior to their inclusion in the study.
Ethics oversight	Informed consent was obtained from all participating patients, and ethical approval was obtained from the Institutional Review Board of Yamaguchi University Hospital (H26-102-7).

Note that full information on the approval of the study protocol must also be provided in the manuscript.
